# The Tomato Genome Encodes SPCH, MUTE, and FAMA Candidates That Can Replace the Endogenous Functions of Their *Arabidopsis* Orthologs

**DOI:** 10.3389/fpls.2019.01300

**Published:** 2019-10-29

**Authors:** Alfonso Ortega, Alberto de Marcos, Jonatan Illescas-Miranda, Montaña Mena, Carmen Fenoll

**Affiliations:** Facultad de Ciencias Ambientales y Bioquímica, Universidad de Castilla-la Mancha, Toledo, Spain

**Keywords:** stomatal development, tomato, *Arabidopsis*, orthologous genes, SPCH, MUTE, FAMA

## Abstract

Stomatal abundance determines the maximum potential for gas exchange between the plant and the atmosphere. In *Arabidopsis*, it is set during organ development through complex genetic networks linking epidermal differentiation programs with environmental response circuits. Three related bHLH transcription factors, SPCH, MUTE, and FAMA, act as positive drivers of stomata differentiation. Mutant alleles of some of these genes sustain different stomatal numbers in the mature organs and have potential to modify plant performance under different environmental conditions. However, knowledge about stomatal genes in dicotyledoneous crops is scarce. In this work, we identified the *Solanum lycopersicum* putative orthologs of these three master regulators and assessed their functional orthology by their ability to complement *Arabidopsis* loss-of-function mutants, the epidermal phenotypes elicited by their conditional overexpression, and the expression patterns of their promoter regions in *Arabidopsis*. Our results indicate that the tomato proteins are functionally equivalent to their *Arabidopsis* counterparts and that the tomato putative promoter regions display temporal and spatial expression domains similar to those reported for the *Arabidopsis* genes. *In vivo* tracking of tomato stomatal lineages in developing cotyledons revealed cell division and differentiation histories similar to those of *Arabidopsis*. Interestingly, the *S. lycopersicum* genome harbors a *FAMA-like* gene, expressed in leaves but functionally distinct from the true *FAMA* orthologue. Thus, the basic program for stomatal development in *S. lycopersicum* uses key conserved genetic determinants. This opens the possibility of modifying stomatal abundance in tomato through previously tested *Arabidopsis* alleles conferring altered stomata abundance phenotypes that correlate with physiological traits related to water status, leaf cooling, or photosynthesis.

## Introduction

Terrestrial plants take up CO_2_ and release water vapor to the atmosphere through stomata, microscopic pores that punctuate the epidermis of leaves and other aerial organs (Ziegler, 1987). Each stoma is delimited by two twin guard cells, whose dynamic shape changes brought about by cell turgor adjustments associated to endogenous and environmental cues open or close the stomata, effectively increasing or decreasing the total pore area (Ziegler, 1987; Raven, 2002). The total surface available for gas exchange depends on the degree of stomatal opening, but also on the number, size, and spatial distribution of stomata across the aerial epidermis. In the last few years, these anatomical features emerged as crucial players in water relations and in photosynthesis and thus in plant growth and reproduction (Dow et al., 2014a; Dow et al., 2014b; Hughes et al., 2017; Devi and Reddy, 2018; Dittberner et al., 2018; Bertolino et al., 2019; Caine et al., 2019). This growing evidence offers a rationale for targeted modification of stomatal abundance aimed at altered physiology useful for crops under particular environments.

Stomata appear gradually during leaf development (Geisler and Sack, 2002; de Marcos et al., 2015). The molecular components regulating this process have been studied in depth in the model species *Arabidopsis thaliana*, providing insight into the networks that control the genetic program of stomatal development (Bergmann and Sack, 2007; Pillitteri and Torii, 2012; Simmons and Bergmann, 2016; recently reviewed by Qu et al., 2017). The links of this program to environmental factors such as light, temperature, atmospheric CO_2_, or water availability have also been explored (Hetherington and Woodward, 2003; Casson and Grey, 2008; Lake and Woodward, 2008; Franks and Beerling, 2009; Casson and Hetherington, 2010; Tricker et al., 2012; Lee et al., 2017; Lau et al., 2018; Meng et al., 2018; Qi and Torii, 2018). The present scenario describes a complex network where membrane receptors and signaling peptides act through protein kinase cascades (to prevent too many stomata and ensure that they are flanked by the needed subsidiary cells to function), modulating the activity of positive drivers of stomata development, the outcome of these opposed regulators determining stomatal abundance and distribution on the leaf epidermis. The key positive regulators are a triad of closely related basic helix-loop-helix (bHLH) transcription factors termed SPEECHLESS (SPCH; MacAlister et al., 2007), MUTE (Pillitteri et al., 2007), and FAMA (Ohashi-Ito and Bergmann, 2006), their transient expression in specific epidermal cell types driving a complex cell division and differentiation process whose outcome is stomata production. These three master bHLHs are collectively referred as SMF proteins and they act in consecutive stages and cell types of the stomatal lineage. SPCH initiates stomatal lineages through the asymmetric division of a protodermal cell that gives rise to a meristemoid and amplifies the meristemoid population by repeated asymmetric divisions (MacAlister et al., 2007). MUTE directs the commitment of the late meristemoid to differentiate into a guard mother cell that will execute a final symmetric division (Pillitteri et al., 2007), and FAMA acts to differentiate the twin cell products into guard cells that compose the stoma and to prevent any further cell division, terminating the life history of this lineage (Ohashi-Ito and Bergmann, 2006). SMFs act together with the bHLHs SCREAM1/ICE1 and SCREAM2 (Kanaoka et al., 2008), and their activity is regulated by phosphorylation involving, among others, YODA-related MITOGEN ACTIVATED PROTEIN KINASE (MAPK) cascades and brassinosteroids-related BRASSINOSTEROID INSENSITIVE 2 (BIN2) (Bergmann et al., 2004; Gudesblat et al., 2012). The main pathway is leaded by the MAPKKK YDA, signaled by peptides of the EPIDERMAL PATTERNING FACTORS (EPFs) family (Torii, 2015) perceived by homo or heterodimers of the membrane receptor TMM (Nadeau and Sack, 2002) and receptor-kinases of the ERECTA family (ERf,; Shpak et al., 2005; Lee et al., 2012). An additional class of peptides from the CLE family and their receptors participate in stomatal development (Qian et al., 2018; Vatén et al., 2018). All these components act in a combinatorial fashion (Lau and Bergmann, 2012; Torii, 2012). This ever increasingly complex scenario (Horst et al., 2015; Qi et al., 2017; Han et al., 2018; Simmons et al., 2019) not only allows stomata to be made, but it also determines stomata distribution and abundance.

Several studies have identified putative orthologs of *Arabidopsis* SMFs in a range of species distributed across the plant phylogeny, from mosses to monocotiledoneous species (reviewed by MacAlister and Bergmann, 2011; Ran et al., 2013; Han and Torii, 2016; Chater et al., 2017; Hepworth et al., 2018). Because most of these studies have an evo-devo approach, they concentrate in scanning the phylogenetic landscape, including model species for hornworts, mosses, ferns, gymnosperms, and, among angiosperms, mostly monocots and basal dicots, adopting *Arabidopsis* as the model for eudicots. Several of these studies include functional assessments of the putative SMFs in various species. In the moss *Physcomitrella patens*, knock-out of the unique functional SMF-like gene present in its genome and phylogenetically close to AtFAMA, rendered stomataless sporophytes, as did the deletion of *PpSCREAM1*, which was essential for stomata formation in contrast with *Arabidopsis* (Caine et al., 2016; Chater et al., 2016). For some monocots, functional studies on putative SMFs are also available. These include complementation assays of *Arabidopsis* loss-of-function mutants with the putative SMF orthologs in rice and maize, which rendered partial positive results and the interesting finding that these grass genomes have duplicated *SPCH*, among other functional differences between the two grasses and *Arabidopsis* (Liu et al., 2009; Wu et al., 2019). This work also established that rice mutants altered in SPCH or FAMA have compromised stomatal development. Further insight in grass stomatal genes came from *Brachypodium distachyon* mutants and showed that the two *BdSPCH* genes act together for normal stomatal production and exhibit mixt *SPCH* and *MUTE* functions (Raissig et al., 2016). In *B. distachyon*, GMC-expressed MUTE drives the symmetric division to form two guard cells but, unexpectedly, it also traffics to neighbor cells to determine stomatal subsidiary cell identity (Raissig et al., 2016).

In dicots, putative SMF orthologs are recognizable in all species studied so far (Ran et al., 2013), but few eudicots have been included in these evo-devo studies and only one of the candidate SMF genes from eudicot crops, the soybean *SPCH* orthologue, has been functionally validated (Danzer et al., 2015). This is surprising, since in monocot crops, it has been well established that stomatal abundance impacts in key physiological processes and this character is therefore a target for breeding (Hughes et al., 2017; Caine et al., 2019). Regarding the potential of SMF genes to modify stomatal numbers, the *Arabidopsis* hypomorphic *spch-5* allele renders a normal-growth phenotype but extremely low stomata abundance, and it is therefore an example of how mutants in this class of proteins could be exploited (de Marcos et al., 2017). Other approaches have studied the physiology of *Arabidopsis* mutants or overexpressors of stomatal development proteins and peptides that modify stomatal abundance (Masle et al., 2005; Tanaka et al., 2013; Dow et al., 2014b; Franks et al., 2015) sometimes concurring with aberrant stomata spacing. In tomato, overexpression of a putative *S. chilense* orthologue of SDD1, a negative regulator of stomatal density, decreased stomatal abundance resulting in a desiccation avoidance phenotype (Morales-Navarro et al., 2018). Genotype-specific expression changes of several stomatal development genes during drought have been described in poplar (Viger et al., 2016). Recently, Papanatsiou et al. (2019) engineered *S. lycopersicum* stomata to undergo faster opening and closure under fluctuating light by expressing the artificial light-activated K channel BLINK, and as a result, growth increased without spending additional water. This pioneer work of optogenetics in plants highlights the relevance of stomata-related processes for improved growth of a dicot crop.

In this work, we aimed at identifying the functional orthologs of the stomatal *SMF* genes in the dicot crop *Solanum lycopersicum*. After selecting the candidates by the amino acid sequence homology of their deduced proteins to *Arabidopsis* SPCH, MUTE, and FAMA, we tested their ability to complement *Arabidopsis* loss-of-function mutants in the three genes, the epidermal phenotypes elicited by their conditional overexpression, and the expression patterns of their promoter regions in *Arabidopsis*. Our results show that the three selected tomato genes encode proteins that carry out the expected functions during *Arabidopsis* stomatal development and that the promoters of at least *SolycMUTE* and *SolycFAMA* are interpreted by the *Arabidopsis* transcriptional machinery and support expression patterns that indicate a similar highly specific activation in specific cell stages during stomatal development. We also identified a *FAMA-like* gene with no apparent function during this process. As the three tomato genes here identified are highly probable master stomatal development regulators, variants identified in available mutant collections or targeted mutations obtained through genome editing could sustain altered stomatal abundance that might exhibit useful changes in water use efficiency or modified cooling capacity without yield penalties.

## Materials and Methods

### Plant Genotypes and Growth Conditions

The *Arabidopsis thaliana* L. (Heyn) wild-type genotype used in this work was Columbia-0 (purchased from NASC, N1092). Mutant lines in Col-0 background were *spch-3* (T-DNA insertion line SAIL_36_B06; MacAlister et al., 2007), *mute-3* (an Ethyl Methane Sulfonate-induced mutant carrying a point mutation; Triviño et al., 2013), and *fama-1* (T-DNA insertion line SALK_100073 T-DNA; Ohashi-Ito and Bergmann, 2006). Tomato plants used in this work were *S. lycopersicum* L. cv. MoneyMaker, purchased from Gartenland GmbH Aschersleben^®^.

For *in vitro* growth, *Arabidopsis* seeds were chlorine gas-sterilized overnight (Clough and Bent, 1998), plated on Petri dishes with solidified growth media as indicated, stratified, and grown at 22ºC and 70% relative humidity with 70 µmol m-2 s-1 of photosynthetically active radiation (PAR) under a long-day photoperiod (16-h light). For soil growth, seeds were sown in preformed 44-mm Jiffy-7^®^ containers, and plants were grown under the same conditions. Tomato seeds were placed on wet filter paper in the dark at 28°C for 48–96 h, transferred to 44-mm Jiffy- 7^®^ containers and grown at 23°C–24°C, 70% relative humidity and 104 μmol photon cm-2 s-1 under a long-day photoperiod.

### RNA Extraction and cDNA Libraries Construction

RNA was extracted from 100 mg (fresh weight) of *S. lycopersicum* 3-day-old (after light exposure) cotyledons from 10 plants. Fresh cotyledons were frozen in liquid nitrogen and RNA was extracted as described (de Marcos et al., 2017) and purified using the Spectrum Plant Total RNA kit (Sigma-Aldrich^®^) and RNase-Free DNase (Qiagen^®^ Nº 79254). RNA quantity and quality in the resultant solution were determined with a NanoDrop ND-1000 spectrophotometer (NanoDrop Technologies^®^). Samples of 1–2 micrograms of purified RNA were reverse transcribed with the High Capacity cDNA Reverse Transcription de Applied Biosystems^®^ kit. Aliquots of the cDNA library were used to selectively amplify the target sequences with *Taq Polimerase HiFi* (Applied Biosystem^®^) and the primers listed in **Supplementary Table 1**. Amplicons were fractionated by agarose electrophoresis, and bands with the expected length purified, sequenced, and used for further cloning.

### Cloning

Cloning was performed through standard procedures using Multisite Gateway^®^ technologies. Each of the *S. lycopersicum* coding sequences for SPCH, MUTE, and FAMA (see **Table 1** for gene identities in iTAG2.40) was cloned in the entry vector PDONR221. The *Arabidopsis* promoters for the three genes were cloned into pDONRP1P4r and in pDONRP2P3r, in which GFP was also cloned. The cDNAs and promoters were recombined into the destination vectors pK7m24GW, or in pK7m34GW to obtain C-terminal translational fusions to GFP. Gene fusions for conditional expression of the tomato proteins were done mobilizing the cDNAs to pH7m24GW, resulting in transcriptional fusions to Olex artificial promoter, and then mobilized to the binary vector pER8GW—which carries the XVE artificial transcription factor under the control of a constitutive promoter (Coego et al., 2014). The promoters of the *S. lycopersicum* genes were defined as the 3,000 bp upstream of their ORFs in GRAMENE. These regions (genomic positions 3:1948998:1951998:1’ for *SPCH*, 1:79169121:79172121:1’ for *MUTE*, and 5:63718221:63721221:1’ for *FAMA*) were synthesized by INVITROGEN, cloned in pDONRP1P4r and mobilized to the destiny vector pGWB404, generating transcriptional fusions to GFP. The putative promoter region of *SolycFAMA-like* (genomic positions 9:70977801:70981100:1) was amplified from genomic DNA extracted using the DNeasy Plant Mini Kit (QIAGEN) (forward primer: 5´-GCACTAGGTATAGCCCTTACAGTC-3´; reverse primer: 5´-TTGTTGATGTTGATAATAATTATAAGAATCATCATCAAG-3´) and cloned into pGWB404 to generate its transcriptional fusion to GFP. **Supplementary Table 2** lists the constructs generated in this work.

**Table 1:** Candidate tomato orthologs for *Arabidopsis SPCH*, *MUTE*, and *FAMA*.

Species	Database	Gene	Gene ID	%ID with AT	% aa ID with AT	Protein length (aa)
**Arabidopsis** **thaliana**	TAIR10	SPCH	AT5G53210	100	100	364
		MUTE	AT3G06120	100	100	202
		FAMA	AT3G24140	100	100	414
**Solanum lycopersicum**	iTAG2.40	SPCH	Solyc03g007410	50.00	54.6	334
		MUTE	Solyc01g080050	64.10	65.8	195
		FAMA	Solyc05g053660	48.56	58.1	383
		FAMA-like	Solyc09g091760	56.28	59.3	247

### Construction and Analysis of *Arabidopsis* Transgenic Lines

*Arabidopsis* transformation was by the floral dip method (Clough and Bent, 1998). The *S. lycopersicum* promoter-GFP and the conditional overexpression fusions were transformed into Col-0. The tomato cDNAs fused to the *Arabidopsis* promoters were transformed into lines heterozygous for the corresponding mutations (*spch-3*, *mute-3*, or *fama-1*). Seeds from these T0 plants were plated on MS medium supplemented with 50 mg/L kanamycin or 25 mg/L hygromycin. T1 antibiotic-resistant plants were genotyped at the *SPCH*, *MUTE*, and *FAMA* loci, and plants homozygous for the loss-of-function alleles were selected. These were self-crossed and the T2 progenies used to identify plants homozygous for the transgenes. Among these, four to eight independent lines were chosen for further studies. Genotyping of *spch-3* and *fama-1* was done by testing with PCR the presence of the T-DNA insertions and the absence of the wild-type alleles; for the point mutation in *mute-3*, a Cleaved Amplified Polymorphic Sequences (CAPS; (Konieczny and Ausubel, 1993) strategy was followed, using *Hph*1. The primers and restriction enzyme used for genotyping are listed in **Supplementary Table 3**.

### Microscopy

For Differential Interference Contrast (DIC), cotyledons were fixed in ethanol:acetic acid 9:1 (v/v) for 16 h, incubated in 90% (v/v) ethanol, and rehydrated in ethanol dilutions 70%, 50%, 30%, and 10% ethanol, and pure distilled water, for 1 h at room temperature per each dilution. Fixed specimens were placed in a chloral hydrate:glycerol:water (8:1:2, w/v/v) clearing solution. Fixed cotyledons were examined with a Nikon Eclipse 90i microscope, and images were recorded with a DXM 1200C camera (Nikon). Cell counts were made with the free software. Confocal microscopy to detect GFP and propidium iodide was done as described (Delgado et al., 2012) with a Leica TCS SP2 confocal microscope. Prior to observation, plants were incubated for 15 minutes in a 10 µg/mL propidium iodide solution (Sigma-Aldrich) to reveal epidermal cell shapes.

### Quantitative Real-Time PCR

RNA for qPCRs was obtained from 50 *Arabidopsis* seedlings for each genotype, collected 3 days after light exposure. Samples from three independent biological replicates were frozen in liquid nitrogen and RNA was extracted with TRIzol (Invitrogen), and column-purified with the High Pure RNA extraction kit (Roche Diagnostics). RNA quality was determined with a NanoDrop ND-1000 spectrophotometer (NanoDrop Technologies). cDNA was synthesized with the High-Capacity cDNA reverse transcription kit (Applied Biosystems) according to the manufacturer’s instructions. The real-time amplification was monitored with the maxima SYBR green qPCR master mix (Thermo Scientific) on a LightCycler 480 II PCR amplification and detection instrument (Roche diagnostics). For the primer sets used for amplification, see **Supplementary Table 4**. Each target gene was paired with two different reference genes (ACT2 [At3g18780] and UBQ10 [At4g05320]). Expression values were calculated with the efficiency method in the LightCycler 480 software version 1.5 (Roche Diagnostics).

### β-estradiol Treatments

Seeds for the conditional overexpression lines were germinated on MS plates containing 10 µM 17-β-estradiol (SIGMA, E8875) as described by de Marcos et al. (2015). At different times after germination, seedlings were examined by confocal microscopy after propidium iodide staining.

### Quantitative Stomatal Traits and Statistics

Organ areas, stomatal index, and density were determined as described by Delgado et al. (2011). To determine SI (number of stomata/total number of epidermal cells × 100), SD (number of stomata per mm), and PCD (number of pavement cells per mm), two 0.4 mm areas adjacent to the median cotyledon axis were scored (modified by Delgado et al., 2011). At least 10 independent plants were used for all determinations. Statistic treatments were done with SPSS vs. 17.0. Index and density data comply with normality according to the Kolmogorov-Smirnov test and variance homogeneity as determined with the Kruskal-Wallis test. Pairwise phenotypic differences were tested statistically by Student’s t-test.

### Sequence Analysis

The deduced protein sequences of *Arabidopsis* SPCH, MUTE, and FAMA were retrieved from the Gramene platform (www.gramene.org) and used to find the tomato proteins with the highest degree of sequence homology through a Pan-taxonomic Compara tree. Deduced protein sequences for the *Arabidopsis* and tomato stomatal bHLH were obtained from TAIR10 and iTAG2.40 and used for a global alignment with the Geneious program (Cost Matrix Blosum90; default settings) in the GENEIOUS R8 platform, to assess the degree of homology. Protein domains and motifs were identified in the current literature and mapped to the deduced proteins.

Phylogenies were constructed with the genetic distance model and the neighbor-joining as statistical method with the Jukes-Cantor program using the Geneious tree builder with a global alignment and the Cost Matrix Blosum90 in the GENEIOUS platform. Deduced protein sequences for the *Arabidopsis* and tomato stomatal bHLHs were obtained from TAIR10 and iTAG2.40.

### *In Vivo* Tracking of Stomatal Lineages

In order to describe the histories of cell division and differentiation events during the development of stomatal lineages in the tomato cotyledon, serial *in vivo* impressions were used, as described by de Marcos et al. (2016). Dental resin impressions of the tomato cotyledon were taken (Light Body Genie; SultanHealthcare), from the earliest stage in which the cotyledon epidermis is accessible after seed germination. Impressions were taken at 24-h intervals, starting at 24-h post light (T1) until 96–120 h (T4–T5) of the same region of the same specimen. Fifty lineages that had produced stomas or CMG at the final time were backtracked up to T1, retrospectively identifying the divisions and cell types (M, SLGC, and CG), recording the type and orientation of each division, cell morphology, and cell fate in later stages. Replicas of the resin impressions were carried out with transparent nail polish, and the replicas were photographed with a Nikon Eclipse 90i microscope with DIC optics connected to a DXM1200C camera.

## Results

### Identification of Putative SPCH, MUTE, and FAMA Orthologs in *S. lycopersicum*

As the three master bHLH stomatal proteins show a recognizable degree of conservation across a broad range of species (MacAlister and Bergmann, 2011; Ran et al., 2013; Chater et al., 2017), we used the *Arabidopsis thaliana* protein sequences to search the GRAMENE (www.gramene.org) database for putative orthologs in the translated *S. lycopersicum* genome, predicted by a Pan-taxonomic Compara tree. For AtSPCH and AtMUTE, the search rendered only one candidate, while for AtFAMA, we found a primary candidate, which we termed SolycFAMA, and a second, shorter protein that was still a putative candidate, which we termed SolycFAMA-like. Table 1 lists gene names and IDs, along with the percentage of nucleotide conservation between each candidate gene and its *Arabidopsis* counterpart and the % of amino acid identity among the deduced proteins.

**Figure 1:**
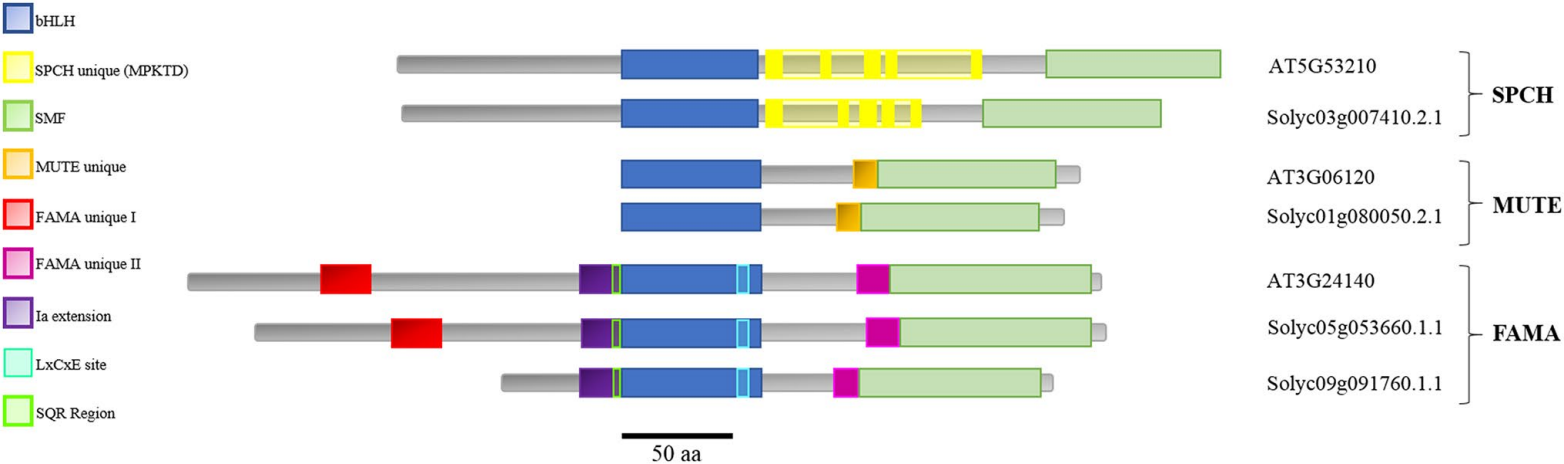
Domain structure of the stomatal SMF proteins in *Arabidopsis thaliana* and the *Solanum lycopersicum* candidates. *Arabidopsis* SPCH, MUTE, and FAMA proteins and their candidate orthologs in tomato are aligned taking as reference their shared bHLH domain (blue). The characteristic SMF C-terminal domain (green) is present in all the proteins. Domains specific for SPCH (yellow), MUTE (orange), and FAMA (unique I in red, unique II in deep purpl, and Ia extension in light purple) are also identifiable in all the proteins, with the exception of FAMA unique I, absent in SolycFAMA-like. The characteristic FAMA SQR putative phosphorylation region and RBR-binding site are shown.

Domain analysis of the candidate proteins (**Figure 1**) showed that tomato SMF proteins displayed recognizable domains described for the *Arabidopsis* proteins (Ran et al., 2013; Davies and Bergmann, 2014). These include the bHLH domain and the characteristic SMF C-terminal domain (green; MacAlister and Bergmann, 2011). Domains specific for SPCH, MUTE, and FAMA (unique I, unique II, and Ia extension) and the SQR putative phosphorylation target (Chater et al., 2017) and the LxCxE RBR-binding motif (Lee et al., 2014; Matos et al., 2014) are also identifiable in the candidate tomato proteins, with the exception of FAMA-unique I, absent in SolycFAMA-like. Domain order and sizes were similar, with some exceptions in SolycFAMA-like that, however, presented most of the relevant FAMA domains.

Sequence comparisons between *Arabidopsis* and tomato for each protein revealed a variable degree of amino acid identity in the different domains (**Supplementary Figure 1**). The bHLH putative DNA-binding domain was remarkably conserved between all the tomato proteins (including FAMA-like) and their *Arabidopsis* orthologs, as described for other species (Ran et al., 2013). SolycSPCH had a slightly shorter N-terminal region that shared a limited identity with AtSPCH, but the SPCH-distinct phosphorylation sites, targets of different signaling cascades during stomatal development and key for protein stability and function in *Arabidopsis* (Lampard et al., 2008; Gudesblat et al., 2012) were almost identical in the tomato candidate orthologue. The SMF C-terminal domain similar in the three *Arabidopsis* stomatal bHLHs was almost identical in their tomato counterparts. The MUTE *Arabidopsis* and tomato proteins shared a high identity in the bHLH region, the MUTE-unique extension, and the SMF domain, albeit not as much as in SPCH and albeit the final amino acid residues at the C-terminus differed. FAMA, the longest of the three proteins, was the most divergent between the two species, except for the bHLH domain and its 5’ extension. The FAMA-unique II domain and the SMF C-terminal domain were also conserved, particularly between *Arabidopsis* and SolycFAMA but also, to a lesser extent, with FAMA-like. The N-terminal half of the three FAMA proteins showed, however, important differences. SolycFAMA shared with AtFAMA the distinct FAMA-unique I domain and other short amino acid motifs (**Figure 1**; **Supplementary Figure 1**). In contrast, FAMA-like lacked most of this N-terminal region, only showing the Ia-extension, a possible SQR motif and some homology stretches immediately upstream from the bHLH domain. FAMA-like showed a high degree of amino acid identity with SolycFAMA in the bHLH and the SMF C-terminal domains, and it harbored the LxCxE RBR-binding motif (Lee et al., 2014; Matos et al., 2014) and a potential phosphorylation site similar to the conserved SQR motif (Chater et al., 2017), as well as the FAMA-unique II region. All these comparisons point that *S. lycopersicum* SPCH, MUTE, and FAMA have potential to be the functional orthologs of the *Arabidopsis* master stomatal regulators. SolycFAMA-like presents a puzzling situation, as it is much shorter than AtFAMA and SolycFAMA and lacks one important N-terminal motif, but its identity is large in other crucial functional FAMA domains.

**Figure 2:**
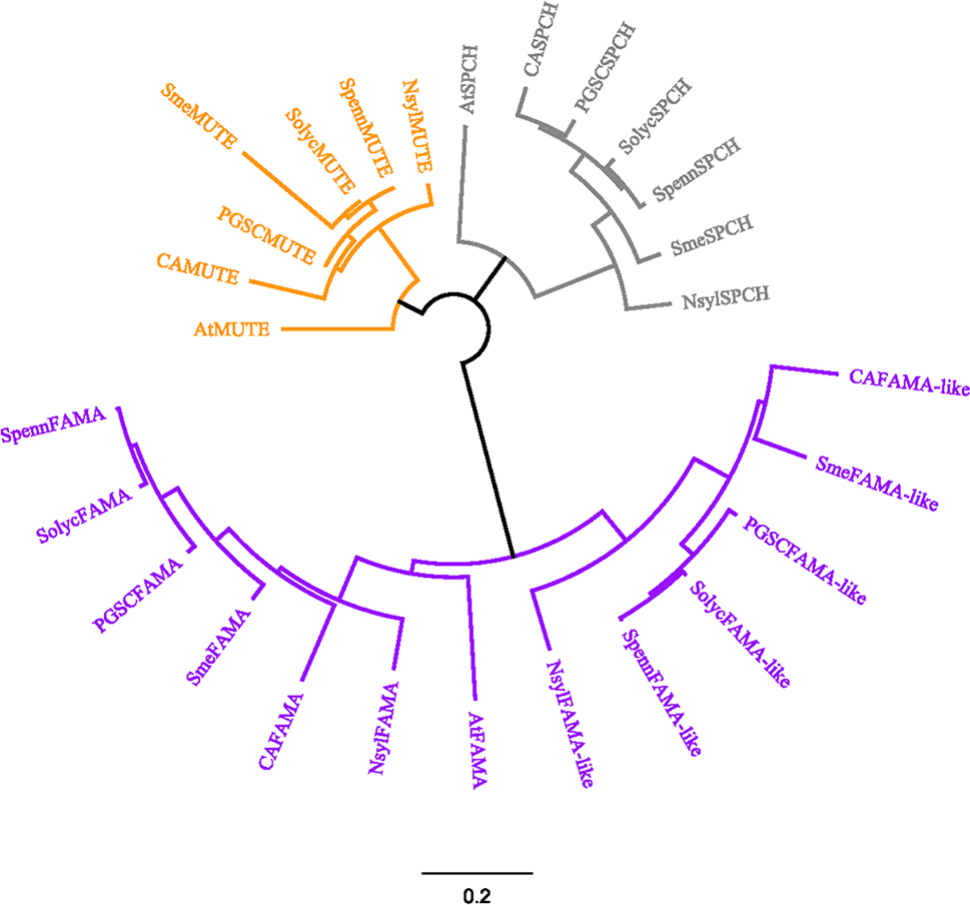
Sequence-based relationships among SPCH, MUTE and FAMA in *Arabidopsis* and *Solanaceous* species. Deduced protein sequences for the *Arabidopsis* SMF and their closest homologs in various *Solanaceous* species were obtained from TAIR10 and iTAG2.40 and used for a global alignment with the Geneious program using Cost Matrix Blosum90 in the GENEIOUS platform. The phylogenetic tree was constructed with the Geneious tree builder. *AT, Arabidopsis thaliana; CA, Capsicum annuum; Nsyl, Nicotiana sylvestris; PGSC, Solanum tuberosum; Sme, Solanum melongena; Solyc, Solanum lycopersicum; Spenn, Solanum pennelli. SPEECHLESS* proteins are in grey, *MUTE* in orange and *FAMA* in purple. Bar, average number of substitutions per site.

To explore to what extent these similarities to *Arabidopsis* are a characteristic of *S. lycopersicum*, we searched other *Solanaceous* genomes for similar proteins, using the SOLGENOMICS database that contains updated sequence information for a growing set of species, and used the retrieved sequences to build a phylogenetic tree. The phylogeny was constructed with the Jukes-Cantor program, using a Geneious tree builder with a global alignment and the cost matrix Blosum90 in the GENEIOUS platform. **Figure 2** shows the phylogenetic relationships for AtSPCH, AtMUTE, AtFAMA, and their putative orthologs in representative *Solanaceous* species (*Capsicum annuum*, *Nicotiana sylvestris*, *Solanum tuberosum*, *Solanum melongena*, *Solanum lycopersicum*, and *Solanum pennelli*). Each of the three stomatal bHLHs grouped together with their putative orthologs, regardless of the species, with SPCH and MUTE showing a close relationship among them. FAMA proteins formed a separate group divided in two distinct clades: AtFAMA was closer to *Solanaceous* FAMA proteins and all FAMA-like proteins clustered together, separated from the FAMA candidates. In all species, FAMA was closer to other species FAMA than to the FAMA-like encoded in its same genome.

Because in *Arabidopsis* the SMF proteins function as heterodimers with ICE1/SCRM1 or SCRM2 (Kanaoka et al., 2008), we asked if the tomato genome harbors sequences putatively encoding these SMF interactors. We used the AtICE1 deduced protein sequence to search for putative orthologs in SOLGENOMICS. The search rendered two candidates (Solyc03g118310.3 and Solyc06g068870.3) coincident with those previously identified as involved in cold tolerance in tomato (previously termed SolycICE1 and SolycICE1-like/ICE1a; Miura et al., 2012; Feng et al., 2013; Kurbidaeva et al., 2014). Protein alignment of both candidates with AtICE1showed a limited overall conservation of the three proteins, although all harbored a bHLH domain, an S-rich motif, and the ACT-domain, with SolycICE1-like protein showing 55% similarity to SolycICE1 and 47% similarity to AtICE1, as described by Feng et al. (2013). It is unclear if any of the two tomato genes represent orthologs of the *Arabidopsis* SCRM1/2 stomatal proteins (see *Discussion*). All these analyses indicated that SolycSPCH, SolycMUTE, and SolycFAMA are the probable orthologs of *Arabidopsis* SPCH, MUTE, and FAMA.

### Expression of Putative Tomato Orthologs

**Figure 3:**
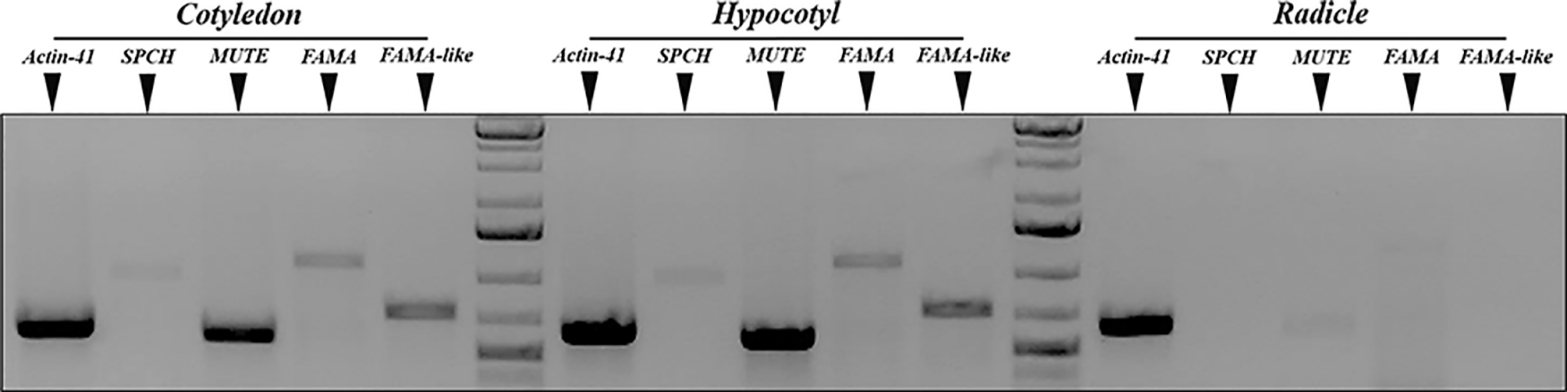
Organ-specific expression of *S. lycopersicum* putative *SPCH*, *MUTE*, and *FAMA* genes. Semiquantitative RT-PCR of 3 dpl cotyledons, hypocotils, and radicles. Primers specific for uniqua regions of *SolycSPCH*, *SolycMUTE*, *SolycFAMA*, and *SolycFAMA-like* were used to amplify the corresponding sequences, and the amplicons fractionated by agarose gel electrophoresis. The normalizer was *S. lycopersicum ACTIN-41*.

In *Arabidopsis*, the three stomatal bHLH genes display a distinct expression pattern, as their transcription is restricted to specific cell stages in the developing stomatal lineages, early during cotyledon development, and they are absent in the mature, fully expanded organ. *AtSPCH* is expressed in dividing meristemoids, *AtMUTE* in late meristemoids and GMCs, and *AtFAMA* in young guard cells, and all these cell types are limited to very early developmental stages of cotyledons and leaves (Ohashi-Ito and Bergmann, 2006; MacAlister et al., 2007; Pillitteri et al., 2007; Triviño et al., 2013). To determine organ-specific expression of the candidate tomato orthologs, we extracted total RNA from 3dpl tomato cotyledons, hypocotyls, and radicles (see *Materials and Methods*) and used the cDNAs to perform gene-specific semiquantitative PCR using the primers listed in **Supplementary Table 1**, designed from the corresponding deduced ORFs from the genomic sequences. **Figure 3** shows the results. Transcripts from *SolycSPCH*, *SolycMUTE*, and *SolycFAMA* were present in cotyledons and hypocotyls, but not in radicles; notably, *SolycFAMA-like* was also expressed only in aerial organs, as the other genes.

We conducted an *in silico* characterization of the expression patters of the tomato stomatal bHLHs taking advantage of the unified RNA-Seq data visualization TomExpress platform (Zouine et al., 2017; http://tomexpress.toulouse.inra.fr/query). Tomato *SPCH* (Solyc03g007410) transcripts accumulate in the shoot apical meristem samples (SAM), particularly at 4dpl and 5 dpl, more strongly in young leaf primordia at 4 and 5 dpl, and only residually in whole leaves, flower parts and incipient ovule walls, overall matching the *Arabidopsis* activity of the *SPCH* promoter. Transcripts are absent in all the rest of the organs listed (roots, seeds, and fruits). Tomato *MUTE* (Solyc01g080050) shows almost undetectable expression, consistent with the transient and cell-stage specific expression reported in *Arabidopsis*. Low expression is found in mature seeds (more than 35 dpa) and mature fruits that should contain mature seeds. Tomato *FAMA* (Solyc05g053660), as expected from *Arabidopsis* data, is expressed at later developmental times in SAM, in leaf primordia and more limitedly in flower parts. Whole leaves show a considerable accumulation of FAMA transcripts, in contrast to SPCH and MUTE and to the *Arabidopsis* data. None of these loci has a gene name, and they are not identified in the platform as putative *SMF* orthologs. Accumulation of *FAMA-like* (Solyc09g091760) transcripts was surprisingly similar to that of *SPCH* and not to *FAMA*, though in general at higher levels, particularly in seeds and young fruits.

Regarding *SCRM/2* candidates, *SolycICE1* (Solyc03g118310.3, not marked in TomExpress as an ICE1-related gene) matches the *SPCH* expression pattern, but it is expressed at much higher levels, notably in SAM and leaf primordia. Solyc06g068870.3 (named *ICE1a* in the platform) was expressed at high levels in seeds and fruits, and more limitedly in all aerial organs. According to the expression pattern, both SCRM candidates could participate in stomata development along with SMFs. None of the genes showed transcripts in roots, with the only exception of a low presence of *ICE1a* transcripts.

Thus, the overall expression profiles of the tomato *SMF* candidates, including *FAMA-like*, and the putative orthologs of *ICE1* are consistent with those expected for stomatal lineage development genes.

### Phenotypic Complementation of *spch-3*, *mute-3*, and *fama-1* by *S. lycopersicum* Candidate Genes

**Figure 4:**
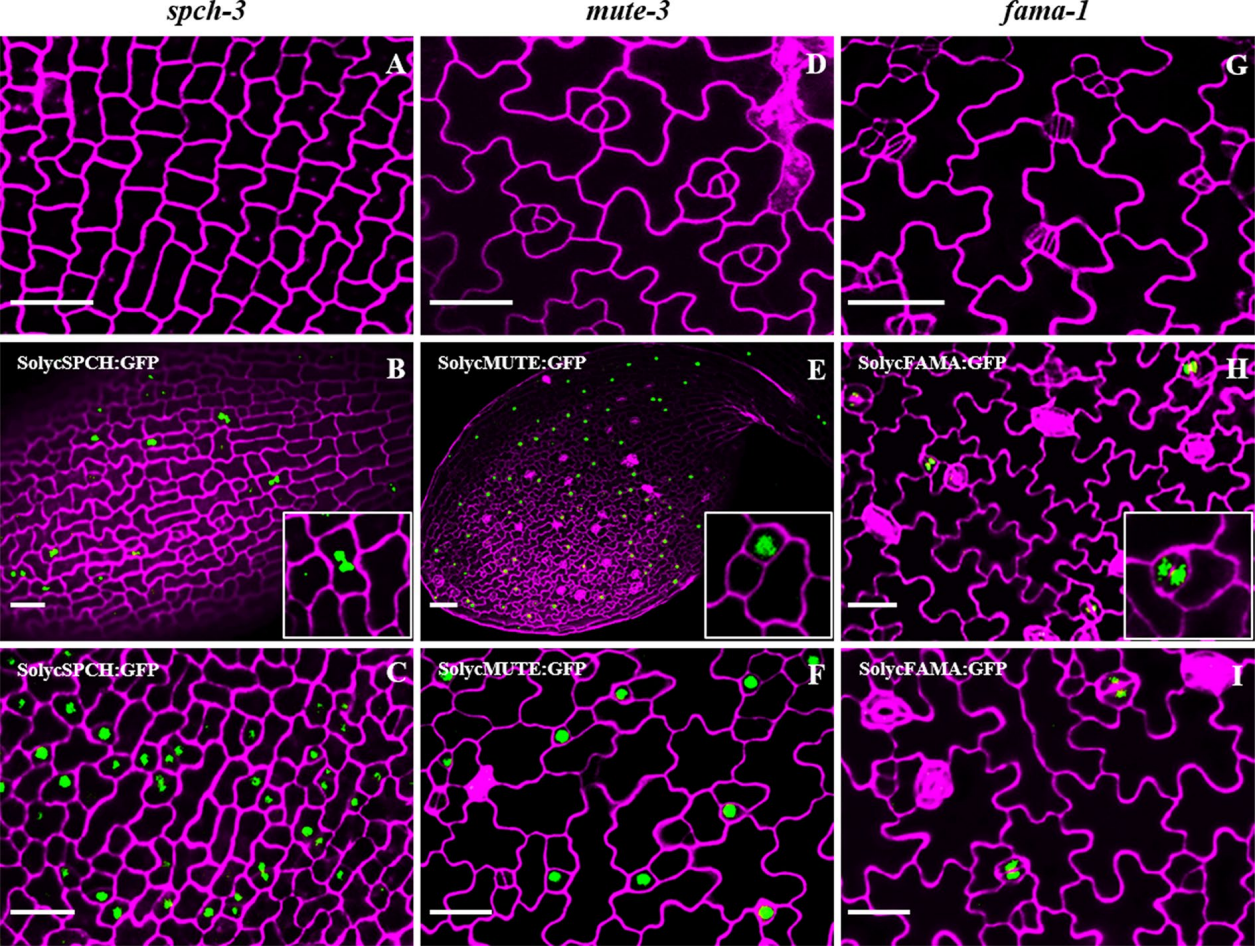
Epidermal phenotypes of *Arabidopsis* mutant lines complemented with tomato orthologs. Confocal cotyledon images of *Arabidopsis* mutants *spch-3* **(A**, **B**, **C)**, *mute-3* **(D**, **E**, **F)** and *fama-1* **(G**, **H**, **I)**. Homozygous mutant plants carried GFP translational fusions to the cDNAs of tomato SPCH **(B**, **C)**, MUTE **(E**, **F)**, and FAMA **(H**, **I)**, under the corresponding *Arabidopsis* promoters. Cotyledons of 3-day-old plants (2 days for **B**, **C**) were stained with propidium iodide to show cell contours (magenta). Note the presence of stomata in the complemented lines and the nuclear accumulation of GFP in specific cell types. Bar: 50 μm.

**Figure 5:**
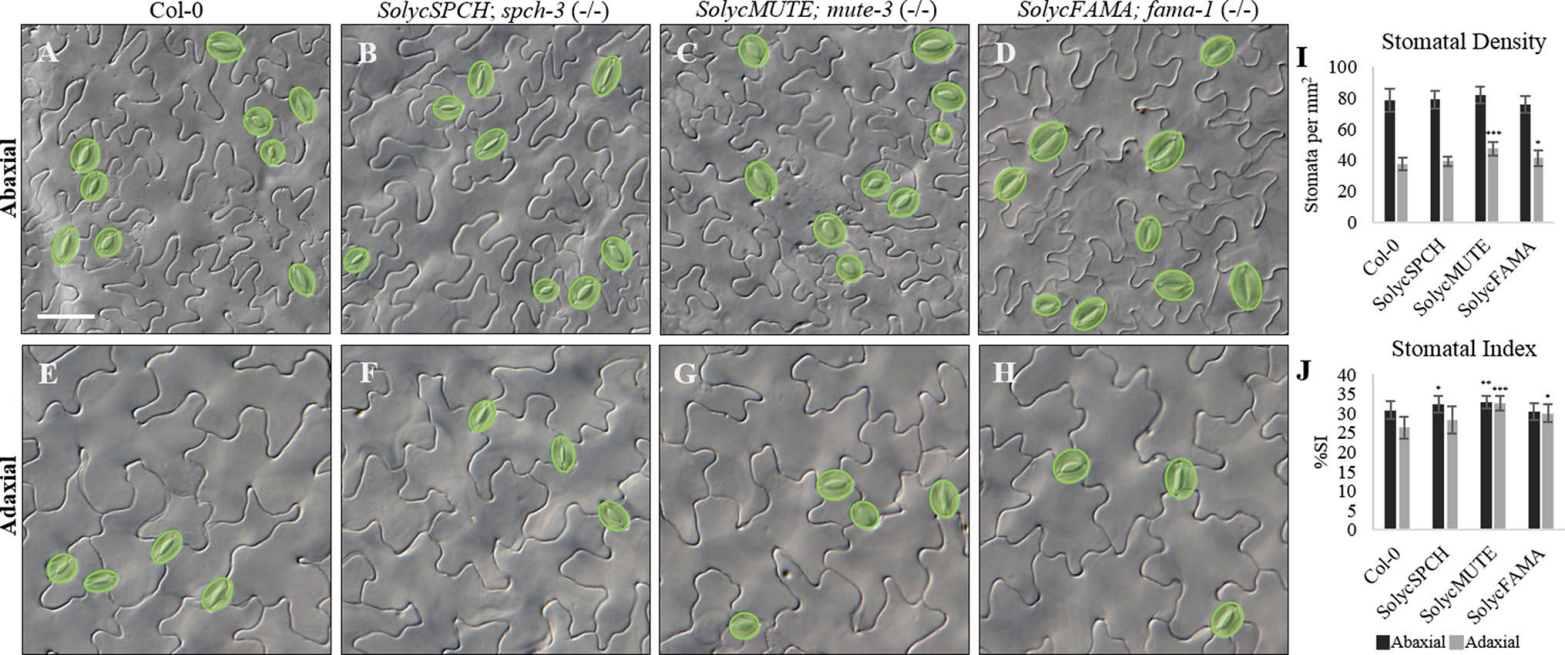
Stomatal Index and Density of the complemented *Arabidopsis* mutant lines. **(A**–**H)** cotyledon DIC micrographs from representative 15-day-old plants of Col-0 and the complemented mutant lines as indicated in the panels. **(A**–**D)** abaxial epidermis; **(E**–**H)** adaxial epidermis. Stomata are false-coloured in green over the micrographs. **(I)** Stomatal Density (number of stomata/mm). **(J)** Stomatal Index (number of stomata/stomata number + pavement cell number), expressed in percentage. Black, abaxial; grey, adaxial. Numbers represented are the medium of 10–20 independent plants. Bars are standard errors. Significant differences by Student’s t-test between each transgenic line and Col-0 are marked (*P < 0.05; **P < 0.001; ***P < 0.000001). Scale bar: 50 µm.

As the deduced tomato proteins identified *in silico* showed a high potential to be the true orthologs of *Arabidopsis* SPCH, MUTE, and FAMA, we proceeded to examine their ability to complement the seedling-lethal phenotypes of the loss-of-function *Arabidopsis* mutants *spch-3*, *mute-3*, and *fama-1*. These mutants were reported to display characteristic stomataless epidermal phenotypes. *spch-3* lacks stomatal lineages, and its epidermis composed of solely pavement cells (MacAlister et al., 2007). *mute-3* produces lineages but it fails to undergo the final stomata-forming steps (Triviño et al., 2013), and *fama-1* also produces lineages but instead of stomata it displays clusters of unpaired guard cells (Ohashi-Ito and Bergmann, 2006). We generated transcriptional fusions of each *Arabidopsis* gene promoter to the cDNA encoding the corresponding tomato protein either by itself or as a C-terminal fusion to GFP (see *Materials and Methods*). All cDNA sequences that we identified corresponded to those described for the reference tomato genome Heinz. Each construct was mobilized to plants heterozygous for each of the stomatal mutant alleles, the resulting plants were self-crossed, and the transgenic progenies screened for the presence of the transgene in homozygosis and for carrying the two mutant alleles at their genomic loci (see **Supplementary Table 5** for a list of transgenic lines selected). In all cases except for those carrying the FAMA-like cDNA, several double-homozygous transgenic lines were selected and epidermal phenotypes examined in four to six of them (**Supplementary Figure 2** shows the genotypes of representative lines at the relevant genomic loci). The confocal images of early developing cotyledons for representative lines in **Figure 4** compare the epidermis of the three loss-of-function mutants with those of the corresponding lines expressing the tomato cDNAs in the mutant genomic backgrounds. SolycSPCH/*spch-3* showed evidence for entry asymmetric divisions at 2 dpl (**Figure 4B**), and at 3 dpl SolycMUTE/*mute-3* (**Figure 4E**), and SolycFAMA/*fama-1* (**Figure 4H**) presented fully differentiated stomata. At later developmental times (**Figure 5**), fully expanded cotyledons of all lines produced apparently normal stomata in their abaxial and adaxial epidermis. These results indicate that the cDNAs of SolycSPCH, SolycMUTE, and SolycFAMA were capable of complementing the defective *Arabidopsis* alleles *spch-3*, *mute-3*, and *fama-1* when expressed in the correct spatial and temporal contexts under the control of the *Arabidopsis* promoter for each of the genes.

### Subcellular Localization of Tomato bHLHs and Expression of *Arabidopsis* Stomatal Genes in the Complemented Lines

As the transgenic lines described above carried protein fusions to GFP, the accumulation of the heterologous tomato SPCH, MUTE, and FAMA could be visualized (**Figures 4B–I**). The three proteins accumulated in the expected cell stages. SPCH-GFP was visible in abundant protodermal cells at very early time points (2–3 dpl), to become progressively restricted to a few cells with meristemoid morphology and disappeared at later cotyledon developmental times (not shown). MUTE-GFP is detected slightly later (3 dpl), in cells with meristemoid or guard mother cell-like morphology, and FAMA-GFP appears in some GMC-like cells and, more prominently as development progresses, in paired guard cells of young stomata. Thus, the tomato proteins were short-lived, as their *Arabidopsis* counterparts, in the cell types determined by the *Arabidopsis* promoters. The three proteins had a nuclear location, indicative of their deduced transcription factor nature. These observations fit with the capacity of tomato SPCH, MUTE, and FAMA candidates to restore stomatal production in the stomataless mutants, suggesting that they integrate in the stomatal development pathway with the other *Arabidopsis* proteins.

Stomatal lineage progression occurs in parallel with expression changes of several genes involved in cell identities and transitions (Adrian et al., 2015; Simmons and Bergmann, 2016). We measured to what extent the heterologous tomato SMF proteins were able to elicit such expression changes in several of these *Arabidopsis* genes (**Supplementary Figure 3**). 3dpl Col-0 seedlings accumulated *TMM* transcripts, indicative of actively dividing stomatal lineages, as well as lower amounts of *SPCH* and *MUTE* transcripts, characteristic of young and late-meristemoids, and guard mother cells, in agreement to previous reports (Simmons and Bergmann, 2016). At this very early stage of cotyledon development, guard cell-specific *FAMA* and *KAT1* transcripts were detected at very low levels, as expected. The expression profiles in the transgenic lines complemented with SolycSPCH, SolycMUTE, and SolycFAMA were similar to those detected in Col-0; *eGFP* expression in each case specifically measured transcripts from the corresponding transgenes, that were absent in Col-0. These results demonstrate that stomatal lineages behaved in terms of specific gene expression similarly in Col-0 and the complemented lines and support the notion that the three tomato proteins we provided can replace the *Arabidopsis* ones in the transcriptional control of stomatal development.

### Quantitative Epidermal Phenotypes of the Complemented *Arabidopsis* Mutant Lines

The qualitative epidermal phenotypes and subcellular protein locations for the candidate tomato proteins indicate that candidate SolycSPCH, SolycMUTE, and SolycFAMA proteins can replace their *Arabidopsis* to determine stomatal lineages development in developing cotyledons. The complemented lines were examined to determine to what extent stomatal abundances in the fully expanded organs compared to Col-0. For that, the abaxial and adaxial epidermes of 20 cotyledons of 15-day-old plants per each type of complemented *Arabidopsis* lines were inspected, micrographs taken, and epidermal cell types (guard cells conforming stomata and pavement cells) counted (**Figure 5**). The apparent stomatal distribution and abundance, as well as pavement cell shapes, were normal in both epidermal sides for all the lines (**Figures 5A–H**). Stomatal densities in all the lines and epidermal sides were compared to Col-0, revealing statistical differences (**Figure 5I**) only for the adaxial side of SolycMUTE and SolycFAMA complemented lines. In all lines, SD was and higher in the abaxial than in the adaxial epidermis, as is typical for *Arabidopsis* cotyledons (Geisler et al., 2000; Delgado et al., 2011). Values for the Stomatal Index, a measure of the proportion of epidermal cells that are stomata and informative of the cell divisions that had taken place in the epidermis, were slightly higher in the abaxial side (**Figure 5J**). In this case, we found statistically significant differences with Col-0 in all the lines. The abaxial SI was moderately higher for SolycSPCH and SolycMUTE lines, while the adaxial SI was higher with a strong significance for SolycMUTE and SolycFAMA. These high abaxial SI corresponded with also significant increases in abaxial SD for the lines complemented with SolycMUTE and SolycFAMA, while the remaining values were similar to Col-0. During the scoring of SI and SD, we searched for aberrant stomatal distribution or differentiation patterns, finding no stomatal clusters, anomalous or aborted lineages or unpaired guard cells. In agreement with these results, the complemented mutant lines grew normally to maturity, flowered, and produced normal numbers of viable seeds, showing wild-type size and general architecture (**Supplementary Figure 4**). These data consistently indicate that the tomato proteins expressed in the transgenic lines display activities that are sufficient to recapitulate the biological effects of their *Arabidopsis* counterparts, although sustained somewhat different stomatal abundances in the mature organ.

### Epidermal Cell Transdifferentiation Mediated by Ectopic Expression of the Tomato Candidate Proteins

**Figure 6:**
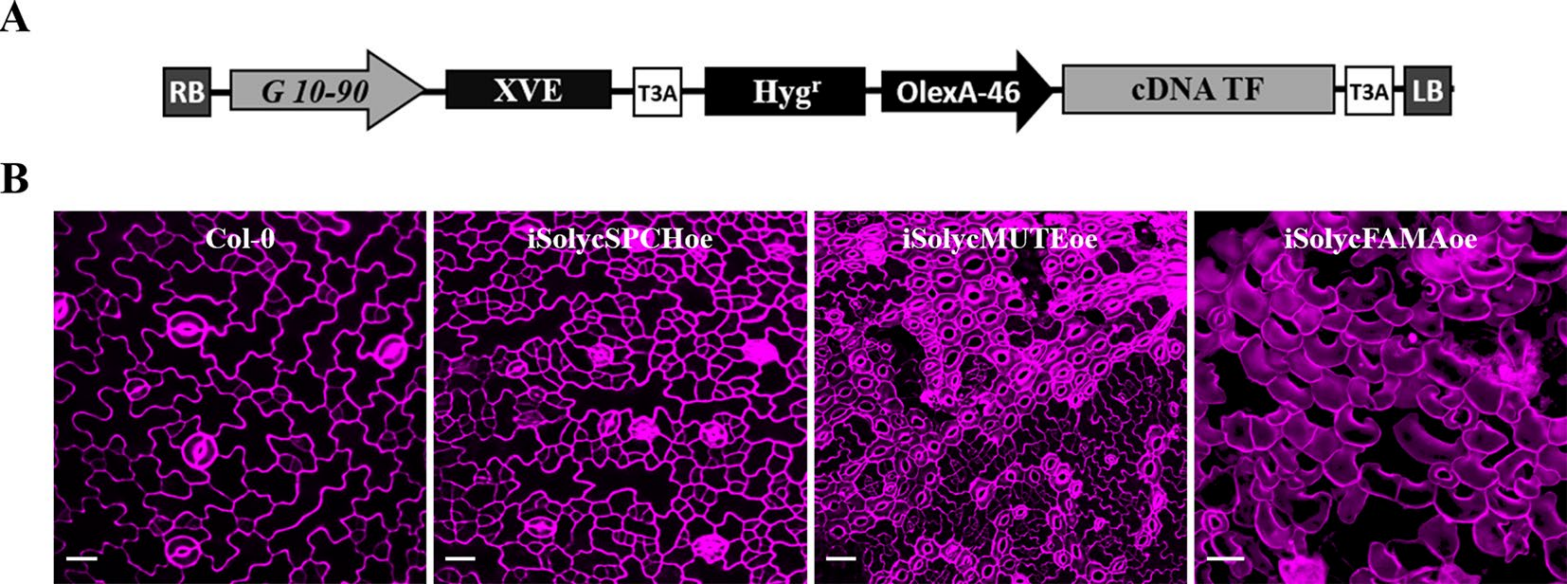
Epidermal cell transdifferentiation induced by overexpression of the candidate tomato proteins in *Arabidopsis*. **(A)** Diagram showing the transgene carried by the conditional overexpression lines. LB, RB: left and right T-DNA borders. G10-90: artificial constitutive promoter. XVE: chimeric transcription factor activated by β-estradiol. OlexA-46: promoter recognized by activated XVE. cDNA: coding sequence for Solyc SPCH, MUTE, and FAMA. T3A: transcriptional terminator. **(B)** Micrographs showing a Col-0 epidermis and the phenotypic effects of overexpressing SolycSPCH (note the small-cell clusters), SolycMUTE (note the only-stomata epidermis), and SolycFAMA (note the unpaired guard cells across the entire epidermis) overexpression. Confocal images of propidium iodide-stained (magenta) 10-day-old cotyledons grown in β-estradiol plates. Bars, 100 μm.

Overexpression of any of the three *Arabidopsis* stomatal bHLH generates distinct phenotypes (Ohashi-Ito and Bergmann, 2006; MacAlister et al., 2007; Pillitteri et al., 2007; Triviño et al., 2013). SPCH overexpression gives raise to extra cell divisions of the meristemoids, resulting in epidermal patches of small cells; MUTE transforms the entire cotyledon epidermis in clustered stomata, with very few discernible pavement cells; and FAMA produces an epidermis entirely composed by unpaired guard cells, as well as guard cell-like mesophyll cells. These phenotypes are interpreted in terms of the ability of SPCH, MUTE, and FAMA to determine cell identity in protodermal cells, interfering with the normal stomatal development program where their expression is constrained to precise and very limited spatial-temporal contexts. To test if tomato proteins conserved such capabilities, we constructed transgenic lines for the conditional overexpression of the four tomato proteins in a Col-0 background. These lines activate the generalized expression of the cDNAs only upon exposure to β-estradiol, overcoming the seedling-lethal phenotypes linked to aberrant epidermis resulting from constitutive overexpression, and they were termed *iSolycSPCHoe*, *iSolycMUTEoe*, *iSolycFAMAoe*, and *iSolycFAMA-LIKEoe*. **Figure 6** shows their epidermal phenotypes as compared to Col-0 when grown on β-estradiol containing plates, each line except for SolycFAMA-like displaying the cell transdifferentiations characteristic of their *Arabidopsis* counterpart, further confirming that the three tomato proteins identified in this work are the functional orthologs of the *Arabidopsis* master stomatal bHLHs.

### SolycFAMA-like Is Not a Functional Orthologue of AtFAMA

**Figure 7:**
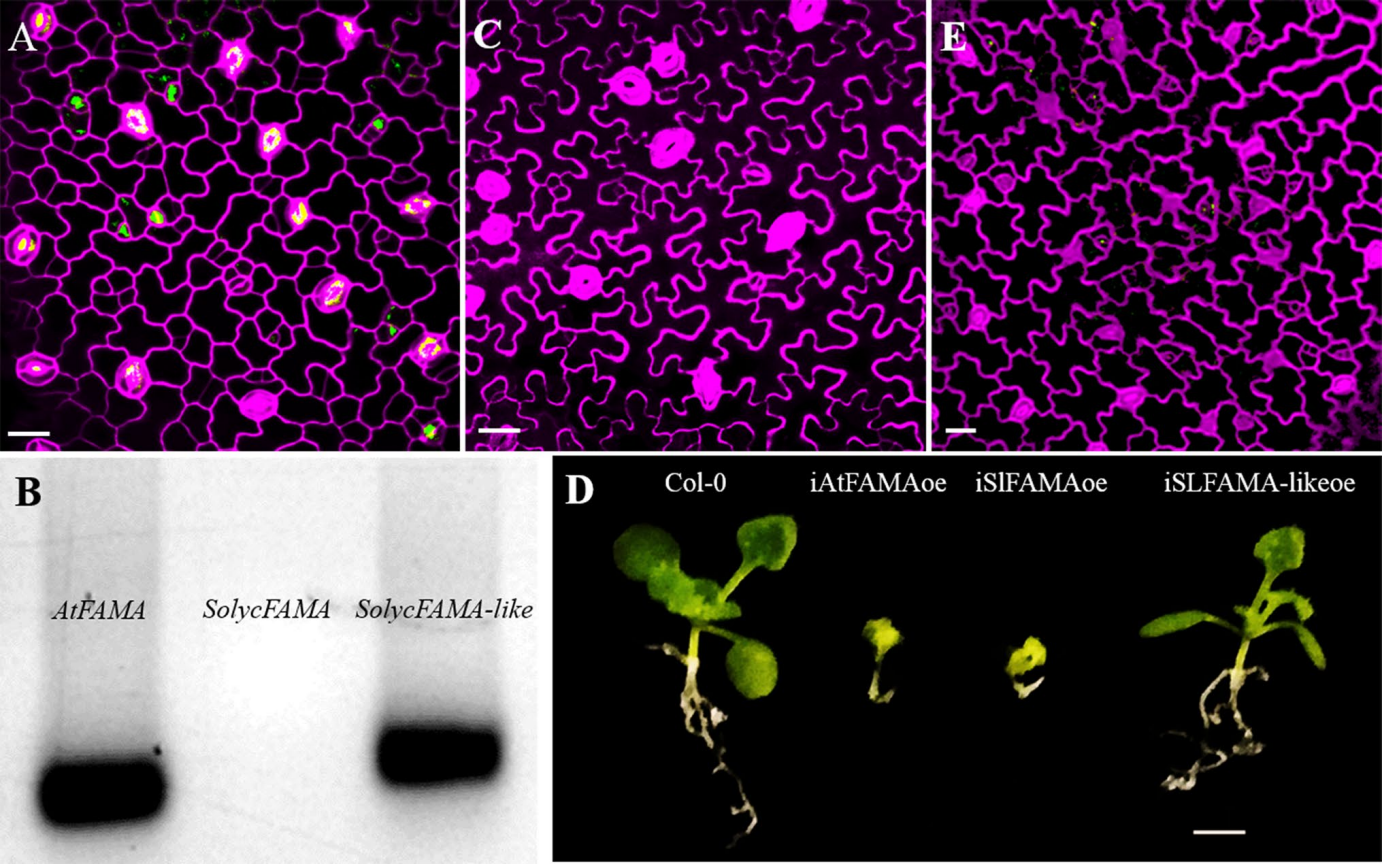
SolycFAMA-like is not a functional orthologue of AtFAMA. **(A)** Confocal image of plants heterozygous for *fama-1* carrying the *AtFAMAp : SolycFAMA-like-GFP* construct. **(B)** RT-PCR in the same lines, using primers for AtFAMA, SolycFAMA, and SolycFAMA-like. **(C)** Epidermal phenotype of *iSolycFAMA-likeoe* line. **(D)** Overall phenotypes of conditional overexpressor lines for AtFAMA, SolycFAMA, and SolycFAMA-like. **(E)** Confocal image of a Col-0 3 dpl cotyledon carrying a *SolycFAMA-like promoter* transcriptional fusion to GFP. Bars: **(A**, **C**, **F)** 50 μm; **(B)** 5 mm.

Our negative results with SolycFAMA-like, which failed to complement the *Arabidopsis fama-1* mutation, suggested that FAMA-like did not express a true FAMA function. All the antibiotic-resistant plants resulting from the transformation of the heterozygous *fama-1* line with the SolycFAMA-like cDNA (**Figure 7A**) were also heterozygous for the genomic mutation at the *FAMA* locus, indicative of a lack of complementation of the lethal recessive *fama-1* mutation. However, such lines specifically accumulated SolycFAMA-like transcripts, but not SolycFAMA, in young cotyledons (**Figure 7B**), indicating that the gene promoter-cDNA fusion functioned correctly. We sequenced the transgene in several of these lines across the entire FAMA-like cDNA-GFP region, confirming the expected sequence. We could also detect GFP in the lines carrying the FAMA-like-GFP translational fusions in some young stomata (as it was driven by the AtFAMA promoter), albeit the accumulation pattern of the fusion protein was somehow inconsistent (**Figure 7A**). The conditional FAMA-like overexpression lines also failed to show the distinct epidermal effects of FAMA overexpression (**Figure 7C**), and the plants grown on β-estradiol did not show the severely stunted phenotype resulting from AtFAMA (Ohashi-Ito and Bergmann, 2006) and SolycFAMA overexpression (**Figure 7D**). Finally, transcriptional fusions to GFP of a 3,000 bp genomic region upstream from the predicted FAMA-like ORF failed to show GFP expression in the expected domains of *FAMA*, although the reporter *Arabidopsis* line occasionally showed some GFP signal in scattered lineage cells (**Figure 7E**). Therefore, SolycFAMA-like does not seem to act as a FAMA orthologue in *Arabidopsis*.

### Expression Domains of the Tomato *SMF* Gene Promoters in *Arabidopsis*

**Figure 8:**
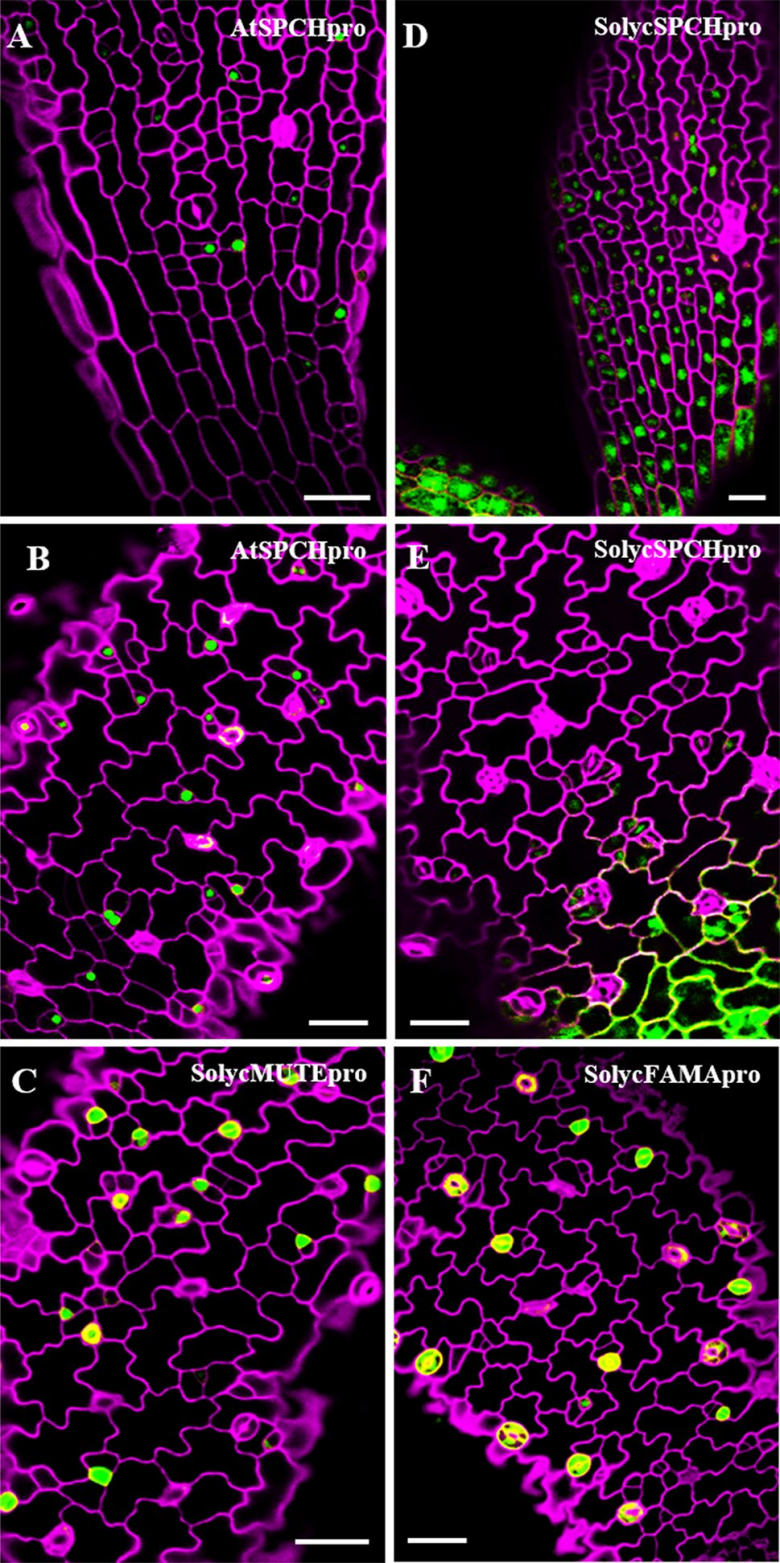
Expression domains for the *S. lycopersicum* promoters of *SPCH*, *MUTE* and *FAMA* in *Arabidopsis*. Confocal cotyledon images of Col-0 lines carrying promoter-GFP transcriptional fusions. **(A**, **B)***AtSPCHpro*; **(D**, **E)***SolycSPCHpro*; **(C)***SolycMUTEpro*; **(F)***SolycFAMApro*. Cell contours were stained with propidium iodide. Cotyledons were from 2 dpl **(A**, **D)** and 3 dpl **(B**, **C**, **E**, **D)** plants. Cell contours were revealed by propidium iodide staining (magenta). Bars: 100 µm.

In *Arabidopsis*, the three SMF stomatal bHLH genes show distinct transcription domains that contribute to restrict their activity to highly specific stages of stomatal lineage cells (MacAlister et al., 2007; Lampard et al., 2009). If the orthologue tomato proteins are to determine stomatal lineage progression in a similar way, the promoters of these genes should also show cell-specific activity, restricted to particular cell types within the lineages. To determine if that was the case, we introduced GFP transcriptional fusions to the promoters (see *Materials and Methods*) of *SolycSPCH*, *SolycMUTE*, and *SolycFAMA* in Col-0 plants and examined their GFP expression patterns in developing cotyledons (**Figure 8**). The *SolycSPCH* promoter sustained a general expression in many protodermal cells at very early stages of seedling development (1–2 dpl), and by 2–3 dpl did not acquire the narrower pattern typical of the *Arabidopsis* promoter, where only some small cells (presumably dividing meristemoids; MacAlister et al., 2007) show GFP accumulation. *SolycMUTE* promoter drove GFP expression at 3–4 dpl only in isolated small cells, sometimes with a rounded morphology (late meristemoids and GMCs), while with the *SolycFAMA* promoter GFP was only visible in paired cells product of symmetric divisions and with the morphology of young guard cells and in some possible guard mother cells. These expression patterns of the tomato promoters matched those described for MUTE and FAMA in *Arabidopsis*, and it was somehow different for SPCH. Therefore, the expression domains of these two tomato genes agree with the conserved protein functions and, together, corroborate the functional orthology of the *S. lycopersicum* genes identified in this work.

### Stomatal Lineage Development in Tomato Cotyledons

**Figure 9:**
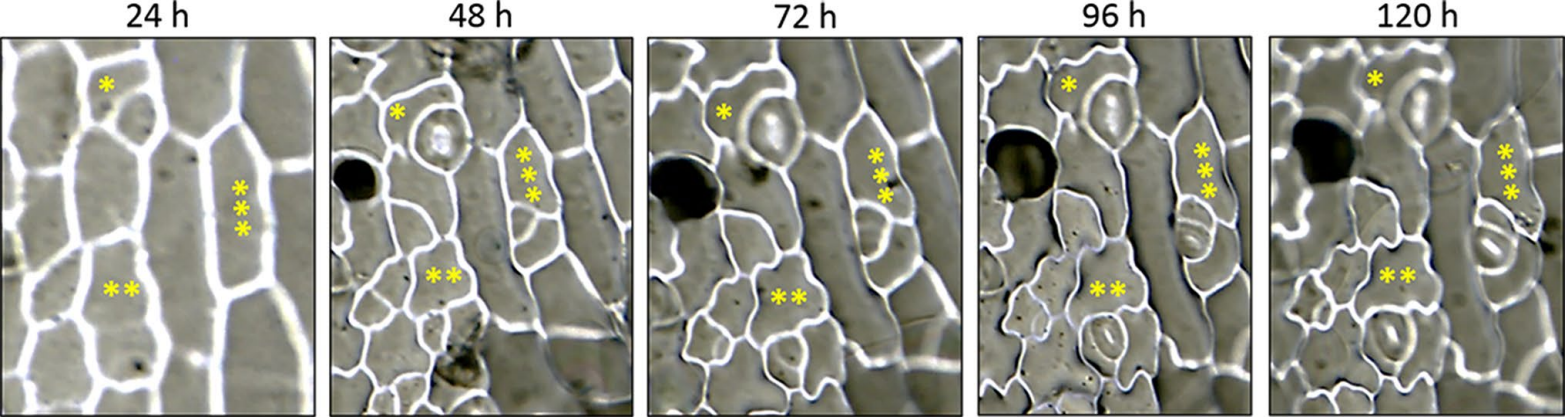
Epidermal cell division and differentiation histories in live cotyledons of *S. lycopersicum*. DIC micrographs of serial impressions replicas taken on the abaxial epidermis of the same cotyledon. Sequential images show the time courses of epidermal differentiation and allow reconstruction of three adjacent lineages experiencing one (*), two (**), or three (***) asymmetric divisions prior to stomata differentiation. Seeds were stratified in darkness for 96 h before their transfer to light conditions. Resin impressions were taken at 24-h intervals along 5 consecutive days.

The consecutive action of SPCH, MUTE, and FAMA at specific cell stages during *Arabidopsis* stomatal lineage development brings about a stereotyped sequence of cell division and differentiation events that culminate with stomata formation (Geisler et al., 2000). Processes intrinsic to the lineages and based on cell-cell communication regulate SMF activity during lineage development (Lampard et al., 2008), and bring about stomatal abundance and distribution patterns in the mature organs that have functional consequences (Vatén and Bergmann, 2012). In tomato, stomata lineage development has not been described. To assess how this process takes place, we carried out a reconstruction of epidermal cell histories by studying *in vivo* serial impressions in incipient cotyledons. We first tracked lineages in cotyledons from seeds stratified 48 h in the dark before transferred to light, taking resin impressions of the same specimen at 24-h intervals up to 120 h. The results (**Supplementary Figure 5**) showed the presence of mature stomata as early as 1 dpl, as well as developing lineages that culminated their development in 96–120 h. Only divisions producing two cell products clearly differing in size, with the smaller cell product presenting the characteristic triangular morphology of meristemoids, were recorded as asymmetric. We reconstructed 50 individual lineages from 10 cotyledons and identified that lineages evolved with one or two asymmetric divisions prior to stoma differentiation. Since in *Arabidopsis* lineages usually evolve with 2–3 asymmetric divisions prior to stoma differentiation, this result suggested a reduction of the number of asymmetrical divisions that could be a characteristic of stomatal development in the tomato cotyledon. However, it could also be an artifact consequence of the tracking method, having lost the first divisions due to the cotyledon not being emerged from the seed coat, what precluded their examination at shorter times. We tested this possibility by increasing the period of dark stratification to 96 h, to take advantage of the slowdown of stomatal lineage development in the dark (Kang et al., 2009; Delgado et al., 2012; Balcerowicz et al., 2014). These conditions made it possible to reconstruct lineages with three asymmetric divisions, as well as abundant lineages with two asymmetric divisions (**Figure 9**). Lineage classification according to the number of asymmetric divisions prior to stomata formation (**Supplementary Table 6**) showed that stomata form after two, three, or one asymmetric divisions of the stomatal precursor, much as in *Arabidopsis*. The overall patterns of stomatal development in anatomical terms were also very similar to those described for *Arabidopsis*.

## Discussion

Work in *Arabidopsis* since the turn of the century established that SPCH, MUTE, and FAMA are the three positive master regulators of stomatal development, together with ICE1/SCREAM1 and SCREAM2. The five proteins are bHLH-type transcription factors, and the first three (termed SMF proteins; MacAlister and Bergmann, 2011) are closely related and drive consecutive stages of stomatal lineage development. In this work, we show that the *S. lycopersicum* genome contains genes whose deduced protein products share a high homology to *Arabidopsis* SPCH, MUTE, and FAMA and are putative candidates for the orthologous SMF genes. The overall domain structure of these proteins and the highly specific motifs unique for each of the three stomatal bHLHs are conserved between *Arabidopsis* and *S. lycopersicum* (this work; MacAlister and Bergmann, 2011; Davies and Bergmann, 2014; Raissig et al., 2016). For SPCH and MUTE, our search identified unique candidates in tomato. The query for *Arabidopsis* FAMA orthologs identified a highly conserved protein, which we termed FAMA, and a second candidate, potentially encoded by Solyc09g091760.1.1. This protein presented a high sequence homology with AtFAMA and with SolycFAMA in the entire C-terminal region starting at the bHLH domain but lacked the entire N-terminal region that harbors the FAMA-unique I motif present in all orthologs identified in several species and in the tomato FAMA protein. We termed this protein FAMA-like. Putative orthologs of FAMA-like are present in all *Solanaceous* species examined but absent from all the other plant genomes. Phylogenetic analysis showed that tomato SPCH and MUTE clustered with the same proteins from other *Solanaceous* species and from *Arabidopsis*. FAMA candidates grouped together separate from SPCH and MUTE and were divided into two clades. FAMA and its corresponding candidates in *Solanaceae* were close to AtFAMA, while *Solanaceous* FAMA-like proteins appeared in a separate cluster. While SolycFAMA, along with SolycSPCH and SolycMUTE, were strong candidates for the tomato SMF triad, FAMA-like could still play some role in stomata development and was therefore studied in parallel to the other three.

Our four candidate genes were transcribed in young tomato cotyledons but not in radicles, and we could clone full-length cDNAs for all of them. SolycFAMA-like transcripts followed a similar organ-specific expression and its cDNA was also cloned and carried along for further analysis. Phenotypic complementation of homozygous loss-of-function *Arabidopsisspch-3*, *mute-3*, and *fama-1* mutants by the tomato candidates was successful, except for SolycFAMA-like, provided that the tomato proteins were expressed under the corresponding *Arabidopsis* promoters. In the young cotyledon epidermis of the complemented lines, cell morphologies indicate overall patterns of cell division and differentiation similar to those described for Col-0, and developing stomatal lineages were identifiable. This indicates that the heterologous proteins, when expressed in correct spatial-temporal frames, behave much as their *Arabidopsis* counterparts and are able to reconstruct the gene circuits altered in each mutant, complementing the stomataless phenotypes and allowing lineage development completion up to full stomata differentiation. The C-terminal GFP fusions to the three tomato SMFs accumulated in the expected cell types where the transgenes should be expressed under the corresponding *Arabidopsis* promoters. All of them showed a nuclear location, as expected for this class of transcription factors. This result demonstrates that the heterologous proteins were not subjected to improper posttranslational modifications that hinder their accumulation or subcellular targeting, but that they accumulated in those epidermal cell types and cell compartment where they could exert their functions replacing their absent *Arabidopsis* counterparts. This is particularly important for SPCH, as protein abundance and activity in *Arabidopsis* are finely regulated by phosphorylation mostly *via* the YDA and the BIN2 kinases (Kim et al., 2012; Gudesblat et al., 2012), and the resulting phosphorylation status of SPCH plays a determinant role in stomatal lineage initiation and in the subsequent asymmetric cell divisions. Our results indicate that the *Arabidopsis* kinase cascades acted upon SolycSPCH, since its GFP fusion shows the expected downregulation as lineages progress: from a broader accumulation in many lineage cells, marking often neighbor lineage cells, it becomes progressively restricted to isolated cells and then it disappears. Failures to downregulate SolycSPCH activity by phosphorylation would have produced a stomata-overproduction phenotype, which was not apparent in our complemented lines (see *below*). These results match with the presence of the target YDA and BIN2 phosphorylation sites in SolycSPCH. Little is known about AtMUTE posttranslational regulation, but in all extent SolycMUTE correctly directs the meristemoid-to-guard mother cell transition in *Arabidopsis*, allowing the symmetric cell division and the subsequent entrance of AtFAMA to produce correctly paired guard cells. Defective SolycMUTE function would have produced extended asymmetric divisions in the lineages, giving rise to a rosette-like phenotype (Pillitteri et al., 2007; Triviño et al., 2013), absent in the complemented lines. Finally, SolycFAMA seems to be targeted correctly by the *Arabidopsis* RBR protein, as a failure in this mechanism that terminates cell divisions in stomatal lineages would have elicited a distinct stoma-in stoma phenotype (Lee et al., 2014; Matos et al., 2014), which we did not detect. The fact that the qualitative stomatal phenotypes of the complemented lines were similar to Col-0, with no aberrant stomatal clusters, SLGC rosettes or improperly differentiated guard cells strongly supports the notion that, overall, the tomato SMFs correctly blended into the *Arabidopsis* protein networks that regulate the processes involved in stomatal development. This is further supported by the fact that endogenous gene expression of master stomatal development regulators correlated with the epidermal phenotypes and was indicative of correct lineage progression. SPCH is the key transcription factor controlling asymmetric cell divisions in stomatal lineages. SPCH positively regulates the expression of itself, *TMM* and *MUTE* among many other genes, in a sequence required for eliciting the complex cell fate and division events that take place in the stomatal lineages (Adrian et al., 2015; Lau et al., 2015; Simmons and Bergmann, 2016). MUTE, in turn, represses *SPCH* and promotes *FAMA* expression to drive the transition of the late meristemoid to a GMC, and to drive its symmetric division (Han et al., 2018). FAMA induces the expression of genes needed to trigger guard cell identity and function, such as KAT1 (Hachez et al., 2011). We found that SolycSPCH supported *AtTMM* and *AtMUTE* expression much as AtSPCH does, providing the molecular explanation of the anatomical phenotypes found in the complemented lines. Similarly, the lines complemented with SolycMUTE and SolycFAMA showed normal expression of the monitored *Arabidopsis* genes. As the SMF proteins act through their interactions with SCREAM/ICE1 and SCREAM2 to drive specific gene expression and lineage development (Kanaoka et al., 2008), our results imply that the tomato SMFs seem able to interact functionally with these *Arabidopsis* co-regulators, as well as with others involved in the transcriptional control of stomatal development. It is unclear if one or the two tomato ICE1 candidate proteins, which show functional similarity with AtICE1 in the responses to cold and other stresses (Miura et al., 2012; Feng et al., 2013) are the true SMF partners during lineage development in tomato plants. It seems that ICE2/SCRM2 appeared in Brassicaceae as the result of a gene duplication of ICE1/SCRM followed by partial neofunctionalization, and other angiosperms have only ICE1 orthologs (Kurbidaeva et al., 2014; Qu et al., 2017). According to TomExpress, both candidate genes are expressed broadly in aerial organs, including young leaf primordia, and could therefore play similar roles in tomato stomatal lineages as SCRM/2 do in *Arabidopsis*.

Stomatal Index and, to a lesser extent, Stomatal Density, are influenced by the history of cell divisions and differentiation events during stomatal lineage development. To examine more in detail to what extent the tomato SMFs quantitatively promote this process, we examined stomatal abundance traits in the mature cotyledons of the complemented lines. All complemented lines showed SIs significantly higher than Col-0, which were particularly high in SolycMUTE-complemented adaxial cotyledon epidermis. Although this could suggest that the tomato proteins were poorer subjects of the *Arabidopsis* negative regulatory networks, there are other possible explanations, such as higher transcription from the transgenes than from the native genes, favoring higher accumulation levels of the heterologous proteins. In most cases, these differences did not translate into SD, and all lines developed normal adult plants that produced fertile seeds. All lines had anatomically normal mature epidermes with interdigitated pavement cells showing the distinct morphology in the abaxial and adaxial sides typical of Col-0.

All these results indicate that *S. lycopersicon* putative SMF proteins behaved very much as their *Arabidopsis* counterparts when expressed in the highly specific cell types and restricted developmental windows determined by the *Arabidopsis* promoters that drive their expression in our transgenic lines. The *Arabidopsis* SMFs display a notable capacity to override endogenous cell differentiation processes when overexpressed in protodermal cells, making these transcription factors strong transdifferentiators that can determine by themselves cell fates in some developmental contexts (Ohashi-Ito and Bergmann, 2006; MacAlister et al., 2007; Pillitteri et al., 2007; Triviño et al., 2013). In the young protodermis, AtSPCH overexpression induces ectopic asymmetric divisions resulting in epidermal patches of meristemoid-like small cells. AtMUTE transdifferentiates most protodermal cells into guard mother cells, producing an all-stomata epidermis and sparse if any pavement cells. Finally, AtFAMA dictates that protodermal cells directly acquire guard-cell fate, skyping the guard mother cell symmetric division and producing an epidermis entirely composed by unpaired guard cells. These morphogenetic capacities are very characteristic of SMFs, and as consequence of their ectopic and continued expression, protodermal cells prematurely acquire cell fates, overriding the stereotyped epidermal development that is determined at large by the specificity of a controlled and partly sequential transient expression of SMF genes. We found that conditional overexpression of the *S. lycopersicum* SMF proteins elicited the same epidermal phenotypes described for *Arabidopsis*, demonstrating that these tomato proteins have the capacity to lead the transdifferentiation of protodermal cells much as their *Arabidopsis* counterparts. Taken together, our results indicate that the tomato proteins studied in this work are the true functional orthologs of *Arabidopsis* SPCH, MUTE, and FAMA. Furthermore, the tomato SMFs seem to interact correctly with the *Arabidopsis* SPCH, MUTE, and FAMA partners, such as SCRM/2, and receive the correct regulatory signals for protein activity, in order to construct normal stomata patterns that sustain normal plant growth and reproduction.

To be considered true functional orthologs of the *Arabidopsis* SMFs, we would expect that *SolycSPCH*, *SolycMUTE*, and *SolycFAMA* genes show a discrete expression pattern restricted in time and space to specific cell stages in the stomatal lineages, as the *Arabidopsis* counterparts do. To test if this was the case, we expressed in *Arabidopsis* transcriptional GFP fusions to the presumed promoter regions (ca. 3,000 bp upstream from the ORFs) of the tomato genes. The SolycSPCH promoter was specifically expressed in the *Arabidopsis* cotyledon epidermis as early as 2 dpl, but in contrast to the *Arabidopsis* promoter (MacAlister et al., 2007), it was not restricted to dividing meristemoids and had a broader expression through most young protodermal cells, failing to show a distinct lineage-specific expression. The reasons for this divergence among *Arabidopsis* and tomato are unclear. The deduced protein from the SolycSPCH gene was shorter in tomato than in *Arabidopsis*, and its aa identity was only 52%, the lowest of the three tomato SMF orthologs, albeit it complemented the *spch-3* mutant, as discussed above.

The promoters of *SolycMUTE* and *SolycFAMA*, in contrast, showed a highly specific expression in the expected cell types and developmental windows. *SolycMUTEpro* was active at early stages of cotyledon development, and only in epidermal cells whose physiognomy corresponded to late meristemoids or guard mother cells, with GFP never detected in the paired guard cells of young stomata, much as *Arabidopsis* MUTE (Pillitteri et al., 2007). Similarly, *SolycFAMApro* mimicked the *Arabidopsis* expression in young paired guard cells and in some cells with the morphology of late meristemoids or guard mother cells (Ohashi-Ito and Bergmann, 2006); again, as expected, mature stomata did not express GFP. This notable similarity between the expression domains of the *Arabidopsis* and the tomato promoters of MUTE and FAMA in *Arabidopsis* plants indicate that the *S. lycopersicum* promoters probably drive expression in similar cell types in tomato plants, but also that their cis regulatory elements are correctly interpreted by the *Arabidopsis* transcriptional machinery. This finding points at a deep conservation between the two species in the key regulatory pathways that impose concurrent and sequential expression of MUTE and FAMA in consecutive cell stages during stomatal development, suggesting that at least the last part of the molecular players of this developmental pathway are very similar in tomato and *Arabidopsis*.

The results obtained from *in vivo* monitoring of epidermal divisions in the cotyledon of *S. lycopersicum* indicate that stomata development follows patterns similar to those described in *Arabidopsis* (this work; Bergmann and Sack, 2007). First, cell lineages are formed that end up producing a stoma after a variable number of asymmetrical divisions. Second, only the smaller sister cell product of the previous asymmetric division, which has a triangular morphology suggestive of meristemoid identity, underwent further asymmetric divisions. Third, the sequential asymmetric divisions often follow a centripetal spiral, placing the stoma surrounded by sister cells that do not differentiate into a stoma. Fourth, in adjacent lineages, the asymmetric divisions were oriented in such a way as to distance the new meristemoids from each other, suggesting that lateral inhibition mechanisms operate to prevent the formation of stomata in contact. All these characteristics match those described for the *Arabidopsis* cotyledons. Thence, cell division and differentiation histories of tomato stomatal lineages are consistent with the high degree of conservation of tomato *SMF* genes with those of *Arabidopsis*. Their ability to complement mutant phenotypes and to mimic aberrant overexpression phenotypes as well as *Arabidopsis* ability to activate transcription from the tomato promoters in specific expected cell types within the lineages support a high degree of similarity in this process at the molecular and anatomical levels in the two species.

The second putative orthologue of AtFAMA in the tomato genome, SolycFAMA-like, is not a functional FAMA orthologue in *Arabidopsis* based in our results. Several lines of evidence support this statement. First, its full-length cDNA did not complement the *Arabidopsis fama-1* phenotype. We obtained several viable *Arabidopsis* transgenic SolycFAMA-like lines after antibiotic selection but genotyping the genomic *FAMA* locus determined that all these lines carried at least one copy of the wild-type *FAMA* allele, suggesting that the homozygous *fama-1* plants were seedling lethal because the tomato transgene gene could not complement the mutation. Supporting this hypothesis, these lines expressed *SolycFAMA-like* but also produced the *AtFAMA* transcript. We sequenced the insert in several transgenic lines and confirmed that the promoter-cDNA and the cDNA-GFP fusions were intact. The transgenic plants expressed GFP in some domains of the *Arabidopsis* FAMA promoter (morphologically identified as late meristemoids and guard mother cell), although the GFP signal did not accumulate consistently in paired guard cells of young stomata, as it did in the SolycFAMA complemented lines. Finally, the conditional overexpression of SolycFAMA-like failed to elicit the distinct epidermal phenotype of iSolycFAMAoe and did not show the characteristic unpaired guard cells of FAMA overexpression. Such overexpression of AtFAMA or SolycFAMA also rendered severely stunted seedlings, a trait that iSolycFAMA-likeoe did not display. All these results indicate that this protein does not play a very relevant role in stomata development or at least not in *Arabidopsis*. We also examined the expression pattern of the SolycFAMA-like promoter in *Arabidopsis*, finding only a faint and inconsistent activity in some lineage cells that did not match those expected for a gene acting on the last stages of stomata development, as the *SolycFAMA* promoter did. However, we detected FAMA-like transcripts in young tomato cotyledons (**Figure 3**) and, according to the TomExpress platform (http://tomexpress.toulouse.inra.fr/query), they accumulate to medium levels in SAM and leaf primordia. We identified homologs of SolycFAMA-like in all *Solanaceous* genomes released in SOLGENOMICS so far, but not in other taxons, suggesting that this is a *Solanaceous*-specific gene. The putative role of the *Solanaceae* FAMA-like proteins is unknown. Expression of an AtFAMA variant lacking the entire N-terminal region upstream from the bHLH domain elicited the overproliferation of small epidermal cells that developed into stomata (Ohashi-Ito and Bergmann, 2006). As this phenotype was interpreted as a cryptic MUTE-like function of this truncated FAMA, the SolycFAMA short protein might as well express a similar capacity, and while our conditional overexpression experiments did not reveal it, we cannot rule out that it is exerted in the context of the tomato epidermis and that FAMA-like participates in stomata development.

Besides having conserved bHLH (including the Ia extension) and SMF characteristic C-terminal domains, as well as the FAMA-unique II motif, FAMA-like proteins had the RBR-binding site and the SQR motif adjacent to the bHLH domain as a potential phosphorylation target proposed to be shared by all true FAMA orthologs (Chater et al., 2017). With this structure, the tomato FAMA-like only lacks the pre-bHLH domain including the FAMA-unique II motif present in most FAMA genes. This situation is similar to other putative FAMA homologs described by Ran et al. (2013) in *Amborella trichopoda*, *Carica papaya*, and *Eucaliptus grandis* all of which lacked the conserved pre-bHLH region. These authors grouped atypical FAMA Angiosperm variants including the *S. tuberosum* one in a clade that included the lycofite *Selaginella moellendorffii* and the Gymnosperm FAMAs, close to the ancient unique SMF gene of mosses (Ran et al., 2013). As the ancestral FAMA proteins do not possess the typical pre-bHLH region typical of Angiosperm, FAMA-like could also represent the conservation of the ancestral gene. Grasses have two *SPCH* genes, both required for stomata formation at least in *Brachypodium* (Raissig et al., 2016), whose origin could be the result of gene duplication followed by neo-functionalization (Raissig et al., 2016; Chater et al., 2017). The origin of the *Solanaceae* FAMA-like could be similarly rooted in a local gene duplication, although we found no evidence of a putative role in stomata development in *Arabidopsis*. Although it cannot be ruled out that the protein has a function in tomato, in our hands, its ectopic overexpression in *Arabidopsis* renders a weak phenotype of plants smaller and paler than Col-0 grown in the same conditions, suggesting some sort of interference with *Arabidopsis* development. The elucidation of FAMA-like functions in *Solanaceae* awaits further experiments that are beyond the scope of the present work.

The three tomato SMFs candidates identified in this work are functional orthologs (and expresologues, except perhaps for SPCH) of their *Arabidopsis* counterparts, suggesting that the basic program for stomatal development in *S. lycopersicum* uses key elements of gene networks seemingly conserved with *Arabidopsis*. This opens the possibility of modifying stomatal abundance in tomato by translating previously tested useful *Arabidopsis* alleles that confer altered stomata abundance phenotypes (such as *spch-5*; de Marcos et al., 2017). Since stomatal abundance and size correlate with physiological traits related to water status, leaf cooling, and photosynthesis, tomato lines with SMF alleles that increase or decrease stomatal abundance might be better equipped to cope with the higher temperatures or the lower water supply in the predicted conditions for the future climate.

## Data Availability Statement

The datasets generated for this study are available on request to the corresponding author.

## Author Contributions

MM and CF conceived and supervised the work and obtained the funding, MM performed the *in silico* work, and AO performed most of the experiments. AM and AO designed the gene fusions with the support of MM. JI-M performed the qPCRs. CF wrote the manuscript with the contribution of MM and AO.

## Funding

This work was supported through grant AGL2015-65053-R from the Spanish Government and through grants PPII10-0194-4164 and SBPLY/17/180501/000394 from the Junta de Comunidades de Castilla-la Mancha to CF and MM. The laboratory received support by UCLM intramural grants and EU FEDER funds. AO and JI-M are recipients of PhD grants from JCCM.

## Conflict of Interest

The authors declare that the research was conducted in the absence of any commercial or financial relationships that could be construed as a potential conflict of interest.
