# Supplementary material for: The Tomato Genome Encodes SPCH, MUTE, and FAMA Candidates That Can Replace the Endogenous Functions of Their Arabidopsis Orthologs
Source: Front Plant Sci. 2019 Oct 29;10:1300. doi: 10.3389/fpls.2019.01300 (PMC6828996; doi:10.3389/fpls.2019.01300)
Supplement: Supplementary file 1 [file DataSheet_1.zip › Supplementary Figure 1.docx]

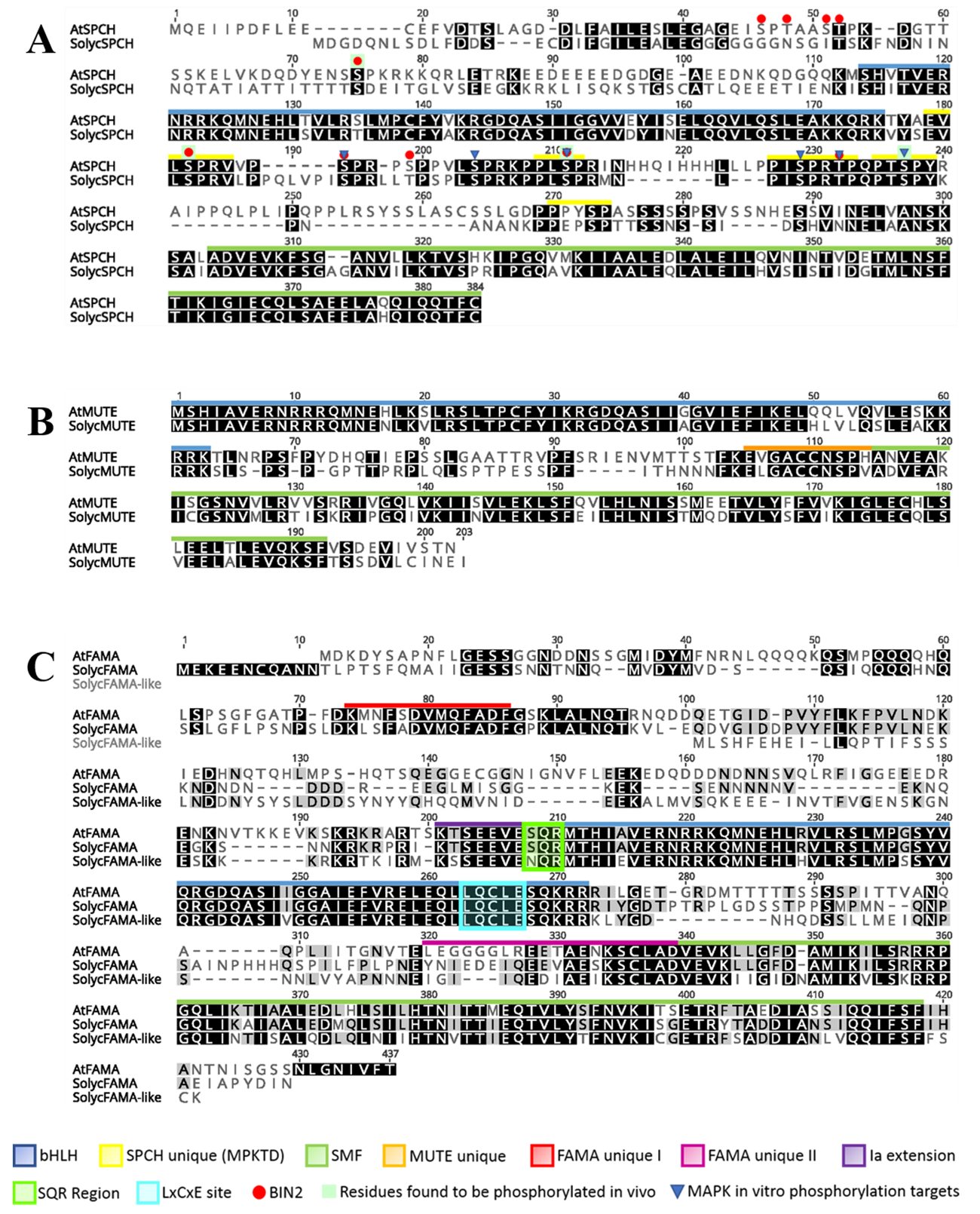


**Supplementary Figure 1. Sequence alignments of the Arabidopsis SPCH, MUTE and FAMA proteins and their putative tomato orthologue**s. Sequence global alignment was performed with the Geneious program using Cost Matrix Blosum90 in the GENEIOUS platform. (**A**) SPCH; (**B**) MUTE; (**C**) FAMA. Conserved residues in all the aligned proteins are boxed in black, and those conserved in two proteins, in grey. Protein domains as described in Figure 1 are marked by coloured horizontal lines above the aminoacid sequences. Domains specific for SPCH (yellow), MUTE (orange) and FAMA (unique I in red, unique II in deep purple and Ia extension in light purple) are also identifiable in all the proteins, with the exception of FAMA unique I, absent in SolycFAMA-like. Phosphorylation targets identified in ATSPCH and the characteristic FAMA SQR phosphorylation region and RBR-binding site are shown.
