# Supplementary material for: The Tomato Genome Encodes SPCH, MUTE, and FAMA Candidates That Can Replace the Endogenous Functions of Their Arabidopsis Orthologs
Source: Front Plant Sci. 2019 Oct 29;10:1300. doi: 10.3389/fpls.2019.01300 (PMC6828996; doi:10.3389/fpls.2019.01300)
Supplement: Supplementary file 1 [file DataSheet_1.zip › Supplementary Figure 2.docx]

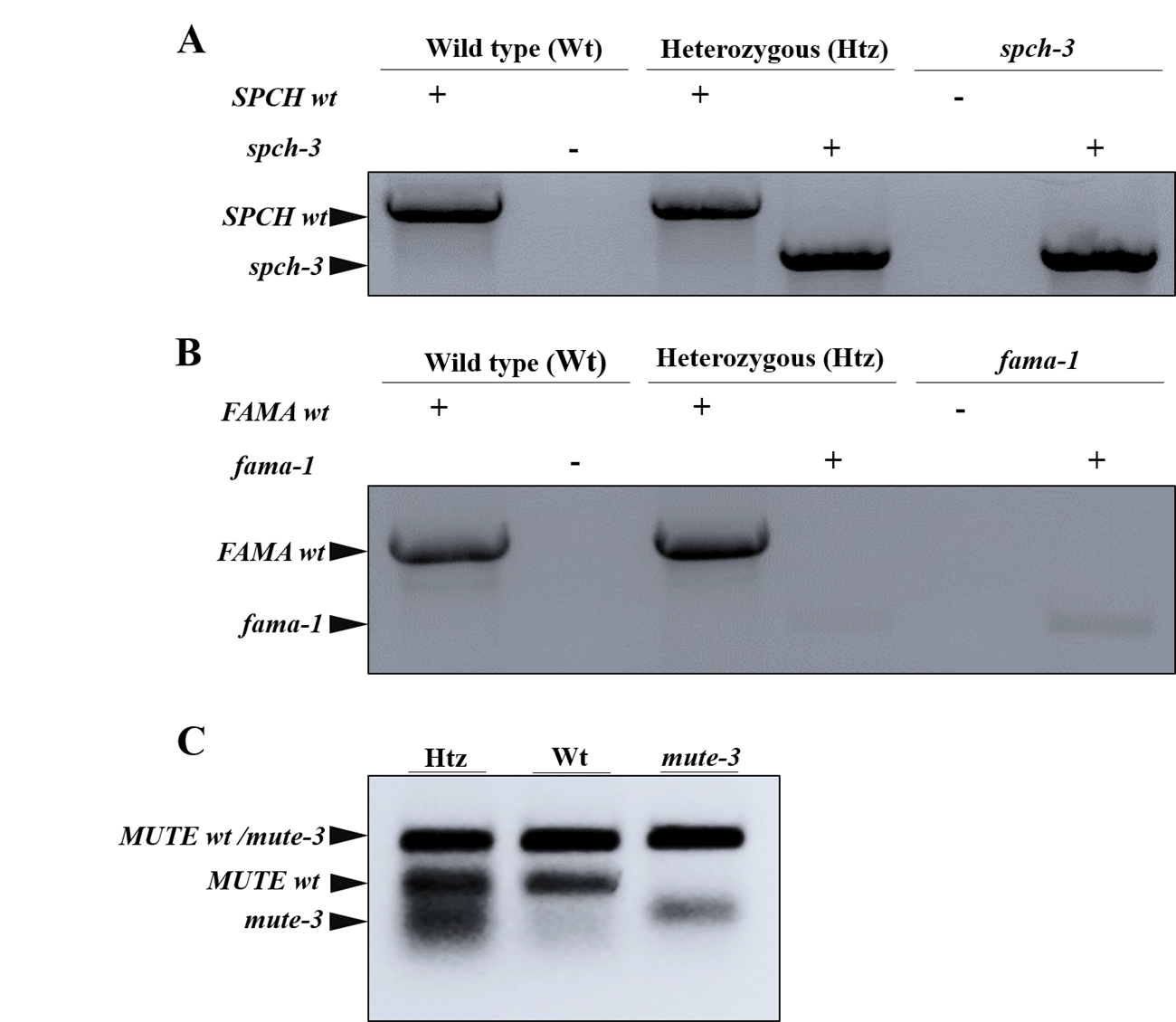


**Supplementary Figure 2. Genotypes of selected transgenic lines at the *SPCH*, *MUTE* and *FAMA* loci.** Agarose gel electrophoresis of the fragments obtained by PCR amplification at the different genomic loci in Col-0 and in representative transgenic lines. (A, B) Diagnostic bands were obtained with allele-specific primers. (C) The amplified fragments were digested with *Hph*l prior to gel fractionation, generating diagnostic allele-specific bands. (A) Genotypes of lines carrying SolycSPCH that were homozygous for *SPCH* or for *spch-3* or heterozygous. (B) Genotypes of lines carrying SolycFAMA that were homozygous for *FAMA* or for *fama-1* or heterozygous*.* (C) Genotypes of lines carrying SolycMUTE that were homozygous for *MUTE* or for *mute-3* or heterozygous.
