# Supplementary material for: The Tomato Genome Encodes SPCH, MUTE, and FAMA Candidates That Can Replace the Endogenous Functions of Their Arabidopsis Orthologs
Source: Front Plant Sci. 2019 Oct 29;10:1300. doi: 10.3389/fpls.2019.01300 (PMC6828996; doi:10.3389/fpls.2019.01300)
Supplement: Supplementary file 1 [file DataSheet_1.zip › Supplementary Figure 3.docx]

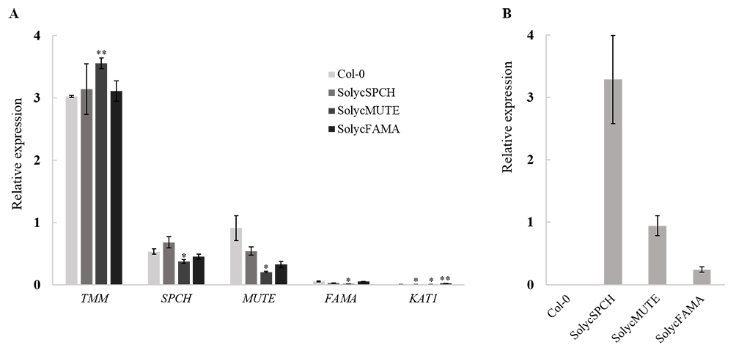


**Supplementary Figure 3. Transcript accumulation in the complemented Arabidopsis lines**. (**A**) Quantitative PCR in Col-0, SolycSPCH-GFP, SolycMUTE-GFP and SolycFAMA-GFP lines to quantify *SPCH*, *MUTE*, *FAMA*, *TMM* and *KAT1* transcripts. (**B**) Transgene-derived transcripts were quantified with with eGFP-specific primers. *UBIQUITIN10* and *ACTIN2* served as reference genes. The results of qRT-PCR were averaged from three independent experiments, with the error bars indicating the SE of the mean. Significant differences by Student’s t-test between each transgenic line and Col-0 are marked (*P<0.05; **P<0.001)
