# Supplementary material for: The Tomato Genome Encodes SPCH, MUTE, and FAMA Candidates That Can Replace the Endogenous Functions of Their Arabidopsis Orthologs
Source: Front Plant Sci. 2019 Oct 29;10:1300. doi: 10.3389/fpls.2019.01300 (PMC6828996; doi:10.3389/fpls.2019.01300)
Supplement: Supplementary file 1 [file DataSheet_1.zip › Supplementary Figure 4.docx]

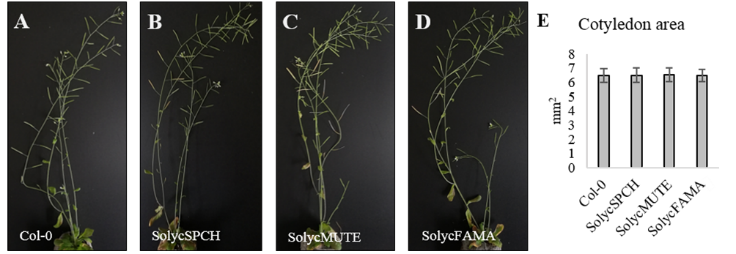


**Supplementary Figure 4. General morphology of complemented Arabidopsis mutant lines.** Representative complemented plants of *spch-3* (**B**), *mute-3* (**C**) and *fama-1* (**D**) were grown to maturity and photographed. (**E**) Fully expanded cotyledons from 10 individual plants per line were collected and their area measured. Students t-tests showed no differences with Col-0 (P>0.05).
