# Supplementary material for: The Tomato Genome Encodes SPCH, MUTE, and FAMA Candidates That Can Replace the Endogenous Functions of Their Arabidopsis Orthologs
Source: Front Plant Sci. 2019 Oct 29;10:1300. doi: 10.3389/fpls.2019.01300 (PMC6828996; doi:10.3389/fpls.2019.01300)
Supplement: Supplementary file 1 [file DataSheet_1.zip › Supplementary Figure 5.pdf]

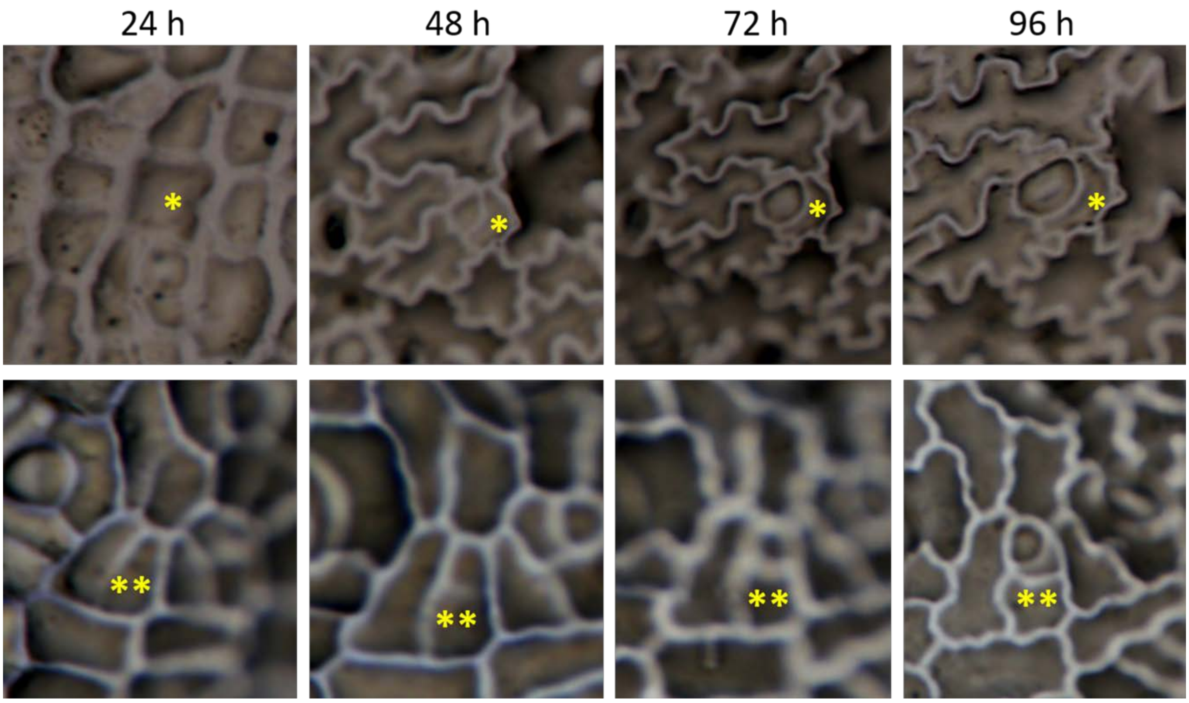

Supplementary Figure 5. In vivo tracking of stomatal lineages in tomato cotyledons. DIC micrographs of serial impressions replicas were taken on the abaxial epidermis of the same cotyledon. The images show the time courses of epidermal differentiation and the reconstruction of stomatal lineages experiencing one (A; *), or two (B; **) asymmetric divisions prior to stoma differentiation. Seeds were stratified in darkness for 48 h before transferring the seedlings to light conditions, and serial resin impressions were obtained at 24 hours intervals
