# Supplementary material for: The Tomato Genome Encodes SPCH, MUTE, and FAMA Candidates That Can Replace the Endogenous Functions of Their Arabidopsis Orthologs
Source: Front Plant Sci. 2019 Oct 29;10:1300. doi: 10.3389/fpls.2019.01300 (PMC6828996; doi:10.3389/fpls.2019.01300)
Supplement: Supplementary file 1 [file DataSheet_1.zip › Supplementary Tables 1-6.pdf]

Supplementary Table 1. Primers used for amplification of tomato cDNAs

Gene	ID Gene	Forward primer	Reverse primer	Size (bp)
Actin-41	NP_001317048	TGGCATCATACTTTCTACAATG	CTAATATCCACGTCACATTTCAT	615
SPCH	Solyc03g007410	ATGGATGGTGACCAAAATTTATCT	TTAGCAGAATGTCTGCTGAATC	1005
MUTE	Solyc01g080050	ATGTCTCACATAGCAGTGGA	CTATATCTCGTTGATACATAAAACATCAG	588
FAMA	Solyc05g053660	ATGGAGAAAGAAGAAAATTGCC	CTAGTTGATATCATATGGGGCTA	1152
FAMA-like	Solyc09g091760	ATGGTTAATATTGATGAAGAAAAGGC	CTATTTGCATGAAAAAAAACTGAATATTTG	744

Supplementary Table 2. Summary of the constructs generated in this work

Promoter	**Coding region**	***Destination Vector***
AtSPCH	SolycSPCH	*pK7m24GW*
AtSPCH	SolycSPCH:GFP	*pK7m34GW*
pG10-90	SolycSPCH	*pER8GW*
AtMUTE	SolycMUTE	*pK7m24GW*
AtMUTE	SolycMUTE:GFP	*pK7m34GW*
pG10-90	SolycMUTE	*pER8GW*
AtFAMA	SolycFAMA	*pH7m24GW*
AtFAMA	SolycFAMA:GFP	*pH7m34GW*
pG10-90	SolycFAMA	*pER8GW*
AtFAMA	SolycFAMA-like	*pH7m24GW*
AtFAMA	SolycFAMA-like:GFP	*pH7m34GW*
pG10-90	SolycFAMA-like	*pER8GW*
SolycSPCHpro	GFP	*pGWB404*
SolycMUTEpro	GFP	*pGWB404*
SolycFAMApro	GFP	*pGWB404*
SolycFAMA-likepro	GFP	*pGWB404*

Supplementary Table 3. Primers used for genotyping

**Primer**	**Sequence 5´-3´**	**Allele identified**
*spch-3*_RP	CACTACCCATTTCTTCC	*SPCH* and *spch-3*
*spch-3*_LP	GAAAAACCTAGATCCTCCCCC	*SPCH*
SAIL_LB	TTCATAACCAATCTCGATACAC	*spch-3*
*fama-1*_RP	CAATACAAAAAGCTCCCCTCAC	*FAMA* and *fama-1*
*fama-1*_LP	TCATTCATTTGCTTCCTACGG	*FAMA*
LbB1	GCGTGGACCGCTTGCTGCAAC	*fama-1*
*mute-3*_RP	CTATAACACCAAATTCCATC	*MUTE* and *mute-3 after Hph*I digestion
*mute-3*_LP	CTAATGTTGTATTGAGAGTTG	*MUTE* and *mute-3 after Hph*I digestion

Supplementary Table 4. Primers used for qPCR

Gene	Forward primer	Reverse primer
Actin-2	TCTTCCGCTCTTTCTTTCCAAGC	ACCATTGTCACACACGATTGGTTG
Polyubiquitin-10	CACACTCCACTTGGTCTTGCGT	TGGTCTTTCCGGTGAGAGTCTT
SPCH	GCTGCTCTTGAAGATTTGGCTCT	TTCTGCACTTAGTTGGCACTCAAT
TMM	GATTGTGTGATGCAGAAACGTC	TCTTCTTCTCATGATAGGTGCTGGA
MUTE	GCTATCTTTTCAAGTTCTTCACCTC	CACTCCAATCCTATCTTAACAACG
FAMA	TCAAGATAACAACTGAAACGAGGT	AATATCTGTTGGATGGAACTTGC
GFP	AAGCAGCAAGACTTCTTCAAGTC	TCGCCCTCGAACTTCACCTC
KAT-1	ACAACAAGTTGCCATAAAGGT	AAGGTGACAAAGCAACAC

Supplementary Table 5. List of selected transgenic lines homozygous for the mutant alleles at the corresponding genomic loci.

Construct	Genotype	Transgenic lines
AtpSPCH::SolycSPCH:GFP	Homozygous (-/-) *spch-3*	1; 8; 5.5; 5.7; 15.6; 37.12
AtpMUTE::SolycMUTE:GFP	Homozygous (-/-) *mute-3*	3; 13; 14; 20; 25
AtpFAMA::SolycFAMA:GFP	Homozygous (-/-) *fama-1*	35; 37; 38; 48
AtpFAMA::SolycFAMA-like:GFP	Heterozygous (+/-) *fama-1*	1;1.1; 2; 2.2; 3

Supplementary Table 6. Classification of stomatal lineages in developing tomato cotyledons

Number of assymetric divisions	Number of lineages (48h darkness)*	%	Number of lineages (96h darkness)**	%
**1**	28	56	5	12
**2**	22	44	27	66
**3**	-	-	9	22

* N= 50
** N= 50
